# Hydrogen spillover through Matryoshka-type (ZIFs@)_*n*−1_ZIFs nanocubes

**DOI:** 10.1038/s41467-018-06269-z

**Published:** 2018-09-17

**Authors:** Guowu Zhan, Hua Chun Zeng

**Affiliations:** 1 Department of Chemical and Biomolecular Engineering, Faculty of Engineering, National University of Singapore, 10 Kent Ridge Crescent, Singapore, 119260 Singapore; 2 Cambridge Centre for Advanced Research in Energy Efficiency in Singapore, 1 Create Way, Singapore, 138602 Singapore

## Abstract

Hydrogen spillover phenomenon is well-documented in hydrogenation catalysis but still highly disputed in hydrogen storage. Until now, the existence of hydrogen spillover through metal–organic frameworks (MOFs) remains a topic of ongoing debate and how far the split hydrogen atoms diffuse in such materials is unknown. Herein we provide experimental evidence of the occurrence of hydrogen spillover in microporous MOFs at elevated temperatures, and the penetration depths of atomic hydrogen were measured quantitatively. We have made Matryoshka-type (ZIFs@)_*n*−1_ZIFs (where ZIFs = ZIF-8 or ZIF-67) nanocubes, together with Pt nanoparticles loaded on their external surfaces to produce atomic hydrogen. Within the (ZIFs@)_*n*−1_ZIFs, the ZIF-8 shell served as a ruler to measure the travelling distance of H atoms while the ZIF-67 core as a terminator of H atoms. In addition to the hydrogenolysis at normal pressure, CO_2_ hydrogenation can also trace the migration of H atoms over the ZIF-8 at high pressure.

## Abstract (web summary)

Hydrogen spillover is well-documented in hydrogenation catalysis, but its existence through metalorganic frameworks (MOFs) remains controversial. Here, the authors provide evidence of the occurrence of hydrogen spillover in microporous MOFs at elevated temperatures, and measure quantitatively the penetration depths of atomic hydrogen.

## Introduction

Hydrogen spillover is a well-known phenomenon in heterogeneous catalysis since the first observation in 1964 on a supported WO_3_/Pt system,^1^ where migration of hydrogen atoms occurs from the metal phase (e.g., Pt) to the support phase (e.g., WO_3_). Generally, hydrogen spillover comprises three connective steps: (i) dissociative chemisorption of molecular hydrogen (H_2_) on a metal catalyst, (ii) migration of atomic hydrogen (H) from the metal surface to the adjacent support, and (iii) hydrogen atoms travel on the surface or throughout the bulk support if it is porous.^2^ Different support materials affect the occurrence of hydrogen spillover significantly. For instance, a recent combined theoretical and experimental study shows that hydrogen atom mobility rate over nonreducible supports (Al_2_O_3_) is about ten orders of magnitude slower than over reducible support (TiO_2_).^3^ Therefore, hydrogen spillover taking place on nonreducible supports is restricted to very short distances. Nevertheless, it can still be observed at elevated temperatures due to a higher kinetic energy.^4,5^

Besides metal oxide supports, hydrogen spillover taking place in metal-organic frameworks (MOFs) has also been studied for hydrogen storage at ambient temperature.^6,7^ Although many studies report that doping MOFs with metal or supported metal catalysts (that is, bridged MOFs) could enhance H_2_ uptakes due to the spillover effect,^8,9^ vigorous debates with respect to the spillover through bridged MOFs continue and in general reproducibility of enhanced H_2_ storage data are not high. It is more likely that the transportation of H atoms in MOFs competes with Eley-Rideal recombination pathways to form H_2_ molecule (viz., H_gas_ + H_ad_ → H_2, gas_).^10–12^ For instance, a weakly chemisorbed H atom in ZIFs could also desorb into a more mobile H atom (which is somehow similar to an H_gas_ species) before hitting the surface to combine with another chemisorbed H atom. In general, there are two possible ways for the H mobility in MOF crystals: (i) diffusion of chemisorbed H, and (ii) diffusion of physisorbed or gas-phase H. Although the physisorbed hydrogen atoms can diffuse freely on graphitic materials,^13^ the theoretical studies suggest that spillover based on this manner would be plugged in an ideal MOF structure.^14^ Moreover, it has been found that diffusion of chemisorbed H in a carboxylate based MOF (*e.g*., IRMOF-1) is still not feasible due to a large energy barrier (up to 1.6 eV) for the migration.^14^ Accordingly, an alternative hole-mediated (due to metal vacancies) hydrogen spillover mechanism in IRMOF-1 has been proposed, which can substantially lower the barriers to enable spillover at ambient condition.^15^ In contrast to the view of spillover for enhanced hydrogen storage, however, some researchers believed that hydrogen spillover effect does not exist on MOF materials at ambient temperature, or the minor enhancement from or by spillover effect is below the detection limit.^10,16,17^ A recent critical review has reported and analysed such an ongoing debate, in which erroneous or irreproducible data regarding spillover in H_2_ storage in the literature have been summarized in detail.^18^

Therefore, to date, it is still unclear whether hydrogen atoms indeed diffuse through the MOF structure, and if they do, how far they could diffuse is then another important question needed to be addressed. In the present study, we design a series of experiments to show direct evidence of hydrogen spillover through zeolitic imidazolate frameworks (ZIFs), a subclass of the most commonly studied MOF materials.^19^ Interestingly, we found that the stabilities of ZIF-8 (based on Zn^2+^) and ZIF-67 (based on Co^2+^) are quite different in the presence of dihydrogen molecules (H_2_) or dissociated hydrogen atoms (H). For instance, both ZIF-8 and ZIF-67 can maintain their structures in an H_2_ environment with temperature up to 300 °C, due to that Zn^2+^ and Co^2+^ have lower standard reduction potentials compared with H_2_ (−0.76 V and −0.28 V, respectively). Since the H atoms are very reactive,^20^ cobalt-containing ZIF-67 framework is susceptible to degradation by the exposure H atoms at a much lower temperature (e.g., 180 °C) while ZIF-8 framework remains almost intact (vide infra). In this demonstrative study, well-defined ZIF-67@ZIF-8/Pt was prepared as an example to investigate hydrogen spillover over the ZIF-8 phase, in which decomposition of the encapsulated ZIF-67 happens only when the hydrogen atoms delivered from the Pt could migrate across the inert ZIF-8 shell and arrive at reactive ZIF-67 core. Herein, Pt nanoparticles with an average size of 3 nm were loaded exclusively on the external {100} facets of the core–shell structured ZIF-67@ZIF-8 nanocubes, noting that the small-sized Pt nanoparticles own high ability of adsorption and dissociation of H_2_ molecule.^21,22^

**Fig. 1:**
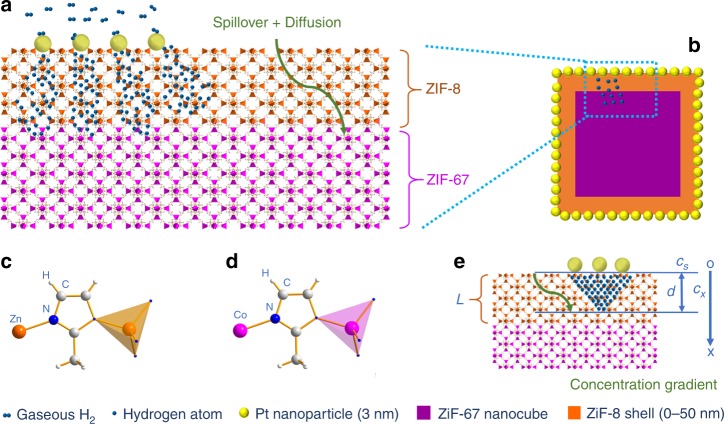
Schematic representation for the hydrogen spillover over ZIF-8. **a** The proposed pathway of hydrogen spillover in ZIF-8, which includes dissociative chemisorption of H_2_ on Pt surface, and subsequent migration of H atoms onto ZIF-8 via spillover and diffusion, finally, hydrogenolysis of ZIF-67 if H atoms arrive the ZIF-67 layer, **b** ZIF-67@ZIF-8/Pt model catalyst in a cross-section view, **c**, **d** the Zn–MeIm–Zn and Co–MeIm–Co linkages the periodic ZIF-8 and ZIF-67 crystals, where the coloured tetrahedrons represent primary building units in ZIFs, and **e** Schematic of concentration gradient of hydrogen atoms and the coordinate system of the model discussed in the main text, where *L* is the shell thickness of ZIF-8, and *d* is the penetration depth of hydrogen atoms

Our methodology is illustrated in Fig. 1. In this process, Pt nanoparticles could dissociate H_2_ molecule into H atoms which then migrate to the adjacent ZIF-8 (as the receptor of H atoms). Since Pt and ZIF-67 are spatially separated, and Pt is located on the external surface of ZIF-8, hydrogen spillover through the ZIF-8 shell (thickness varied between 0 nm and 50 nm) is required for boosting the decomposition of ZIF-67 (viz., hydrogenolysis). In this regard, the ZIF-8 shell serves as a ruler to measure the farthest travelling distance possible of split H atoms while the ZIF-67 core as a terminator of H atoms in this spatial measurement set-up. As the shell thickness of ZIF-8 increases (viz., increasing distance from Pt to ZIF-67), atomic hydrogen concentration decreases dramatically. Thus, on identifying the extent of ZIF-67 decomposition via in-situ gravimetric measurement (under flowing H_2_) combined with *ex-situ* morphological/structural characterizations, we are able to qualitatively measure the concentration of atomic hydrogen arriving at the reactive core of ZIF-67 phase and thereby the penetrating depth of H atoms.

## Results

### Preparation of Matryoshka-type (ZIFs@)_n−1_ZIFs

**Fig. 2:**
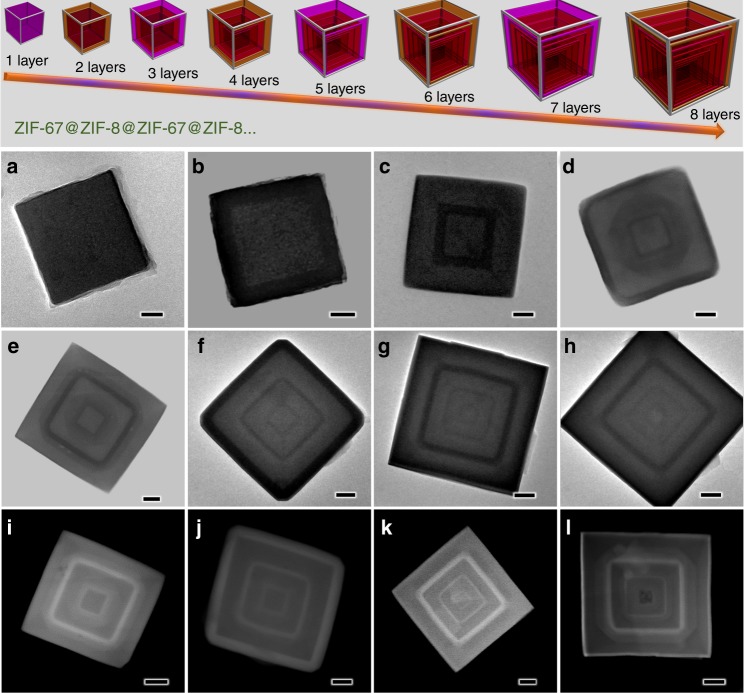
TEM and HAADF-STEM analysis of nano-Matryoshka structured ZIFs. **a** ZIF-67, **b** ZIF-67@ZIF-8, **c** tri-layered ZIF, **d** tetra-layered ZIF, **e**, **i** penta-layered ZIF, **f**, **j** hexa-layered ZIF, **g**, **k** hepta-layered ZIF, and **h**, **l** octa-layered ZIF. The top panel illustrates the synthetic strategy for Matryoshka-type (ZIFs@)_*n*−1_ZIFs with their 3-dimensional geometrical models. Colour code in the models: purple represents ZIF-67 and brown represents ZIF-8. Scale bars in **a**–**l** are 40, 60, 100, 100, 150, 200, 300, 300, 200, 200, 300 and 300 nm, respectively

To ensure identical diffusion pathways of hydrogen atoms starting from the different external surface on ZIFs, it is important to fabricate the highly symmetrical ZIFs structure such as nanocubes since they were bordered by six identical {100} surfaces. Considering that the two representative ZIFs (ZIF-8 and ZIF-67) are isostructural which share the same organic linker (2-methylimidazolate, MeIm), the same crystallographic features (space group$\,I\bar 43m$, *a*_o_ = 16.881 Å in ZIF-8 and 16.908 Å in ZIF-67), but different metal nodes (Zn^2+^ in ZIF-8 and Co^2+^ in ZIF-67, the 4-coordinated Zn^2+^ and Co^2+^ ions are 74 pm and 72 pm in size, respectively), it enables us to combine the two isostructural ZIFs with atomically perfect heterojunctions via multiple heteroepitaxial growth. Firstly, ZIF-67 and ZIF-8 nanocubes were attained respectively by using a surfactant mediated method at room temperature. In our case, surfactant cetyltrimethylammonium bromide (CTAB) molecule functions as a capping agent via interaction between the long hydrocarbon chains and the imidazole linkers in ZIF nodes. It was reported that interaction energies of CTAB with {100}, {110} and {111} facets in ZIF-8 were −775, −395 and −104 kcal/mol, respectively.^23,24^ This implies that CTAB molecules mainly adsorb on {100} facets, and slow down the growth rate of {100} facets, leading to the formation of cubic ZIFs exposing with six identical {100} facets. Subsequently, we prepared core–shell structured ZIF-8@ZIF-67 and ZIF-67@ZIF-8, and other Matryoshka-type (ZIFs@)_*n*−1_ZIFs (e.g., tri-, tetra-, penta-, hexa-, hepta- and octa-layered ZIFs) by stepwise (batch-wise) liquid-phase epitaxial growth. In brief, to construct the (ZIFs@)_*n*−1_ZIFs (where ZIFs = ZIF-8 or ZIF-67; *n* = n^th^ ZIFs, *n* = 1 to 8), we first prepared the first ZIFs cubes (i.e., *n* = 1) and used the sample as core crystals in a fresh solution containing metal ions, MeIm linker, and CTAB surfactant. Under the crystallisation conditions, a new ZIFs layer (*n* = 2) favoured heterogeneous nucleation in the presence of cores (*n* = 1), and a vertically epitaxial growth on top of the core facets spontaneously happened given that ZIF-8 and ZIF-67 have matched lattice parameters.^25^ According to transmission electron microscopy (TEM) and high-angle annular dark-field scanning TEM (HAADF-STEM) observations (Fig. 2 and Supplementary Figs. 1–11), all the produced Matryoshka-type (ZIFs@)_*n*−1_ZIFs preserve cubic shape and the edge length discretely increase as the growth of more shells (2100 nm for the octa-layered ZIFs, shown in Supplementary Fig. 12), explicitly confirming that the shells were grown in an epitaxial manner. In the HAADF-STEM images, heavier contrast of ZIF-8 than that of ZIF-67 was found due to the difference in atomic number of the metal ions (Zn^2+^ vs Co^2+^). In addition, Zn/Co mixed ZIF nanocubes can be obtained by mixing the Zn/Co metal salts together with MeIm linker, in which uniformly distributed Zn and Co were found according to their input ratio (e.g., a molar ratio of 1:1, Supplementary Fig. 13).

### Structural characterizations

**Fig. 3:**
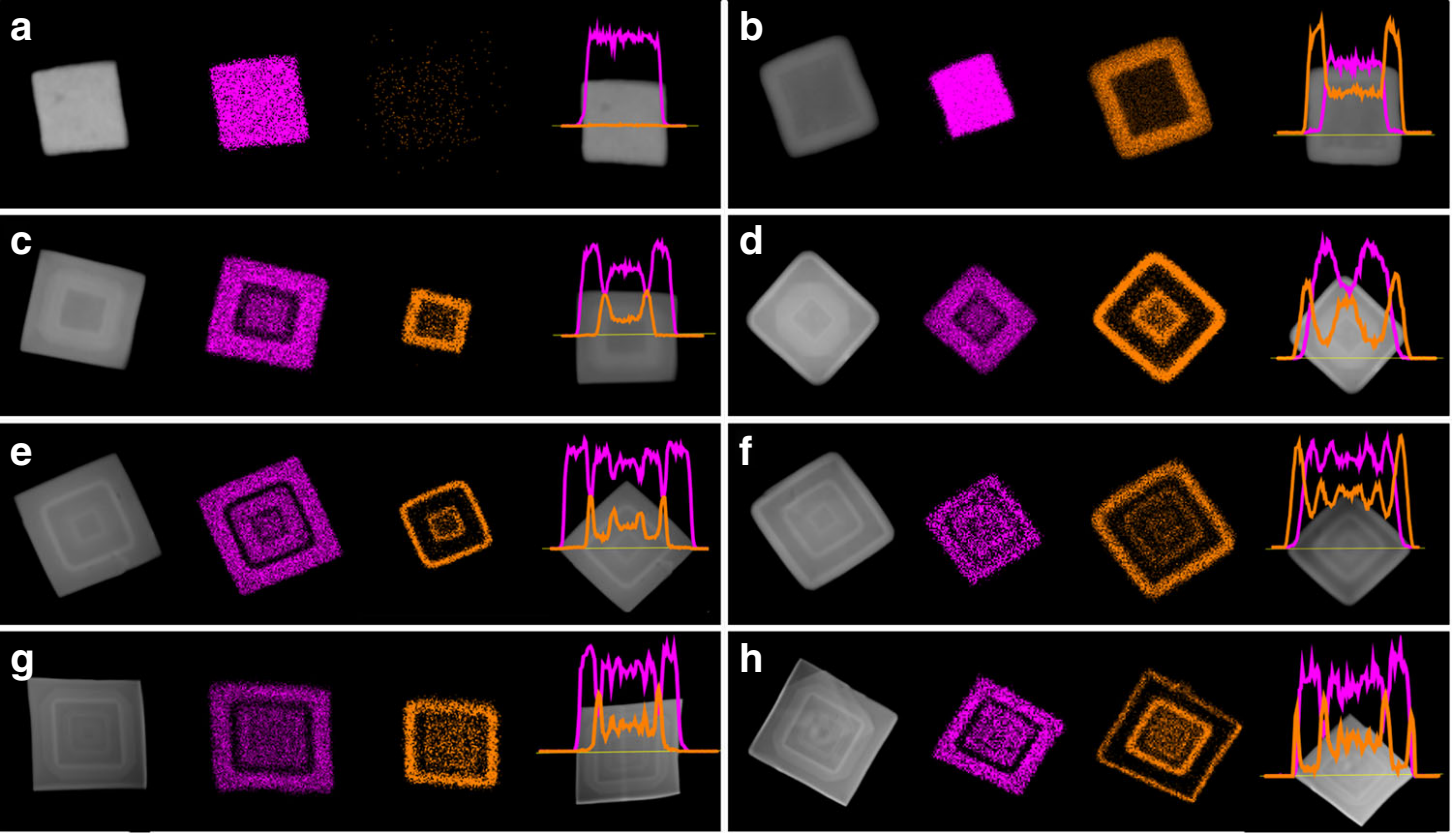
EDX elemental mapping and line scanning of ZIF-67 and nano-Matryoshka structured ZIFs. **a** ZIF-67, **b** ZIF-67@ZIF-8, **c** tri-layered ZIF, **d** tetra-layered ZIF, **e** penta-layered ZIF, **f** hexa-layered ZIF, **g** hepta-layered ZIF and **h** octa-layered ZIF. Colour code: purple represents cobalt; brown represents zinc

**Fig. 4:**
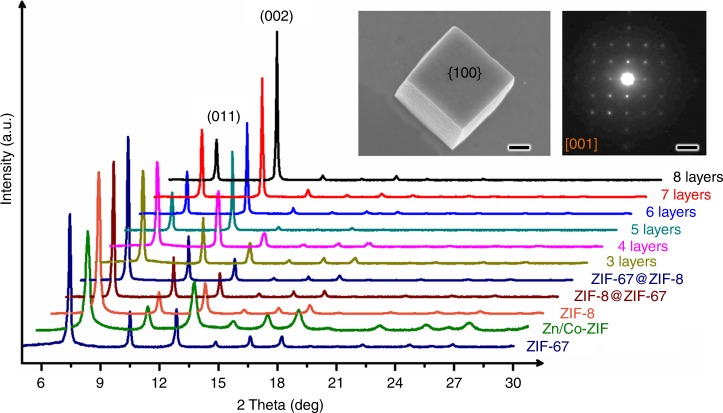
XRD patterns of ZIF-67, ZIF-8, Zn/Co-ZIF, and nano-Matryoshka structured ZIFs. Inset is a SEM image of octa-layered ZIFs (scale bar, 500 nm) and a SAED pattern of the cubic ZIF-67 (scale bar, 1 nm^−1^). See the detailed explanation of SAED information in Supplementary Fig. 15

The topological features of Matryoshka-type ZIFs were clearly confirmed by the corresponding EDX elemental mapping and line scanning in Fig. 3, which provide direct evidence that zinc layers (viz., ZIF-8, highlighted in brown) and cobalt layers (viz., ZIF-67, in purple) are present alternately along the radial direction. An additional proof of the full coverage of a new layer ZIF on the cores can be given by the X-ray photoelectron spectra (XPS). For instance, no Co signal was found if the outmost layer of the composite is ZIF-8. Likewise, no Zn signal was observed if the outmost layer is ZIF-67 (see Supplementary Table 1), which must validate the full epitaxy growth of Matryoshka-type ZIFs. Importantly, the batch synthesis of both ZIFs cubes (*n* = 1) and Matryoshka-type (ZIFs@)_*n*−1_ZIFs can be conducted on a gram scale or larger without compromising the quality, in which the products are still in fairly homogeneous particles as seen from Supplementary Fig. 14. The cubic-phase crystalline structures of ZIF-67 and ZIF-8 have also been verified by XRD measurements, as shown in Fig. 4. All diffractograms are characteristics of the typical ZIFs, which reveal that the crystalline structures of all the studied ZIFs were staying unchanged, despite the growth of more shells. Interestingly, discrepancies in the intensity ratio of (002) to (011) are found, which suggests that Matryoshka-type (ZIFs@)_*n*−1_ZIFs with increasing size exhibit facet-to-facet ordering with the (002) plane (*viz*., the faces of all these product cubes) being parallel to the XRD silicon wafer (i.e., sample holder).^26^ Furthermore, the {100} oriented facets of the ZIF-67 single crystal are also confirmed from the SAED (Fig. 4 (inset) and Supplementary Fig. 15).

### Fabrication of ZIFs/metal nanocomposites

**Fig. 5:**
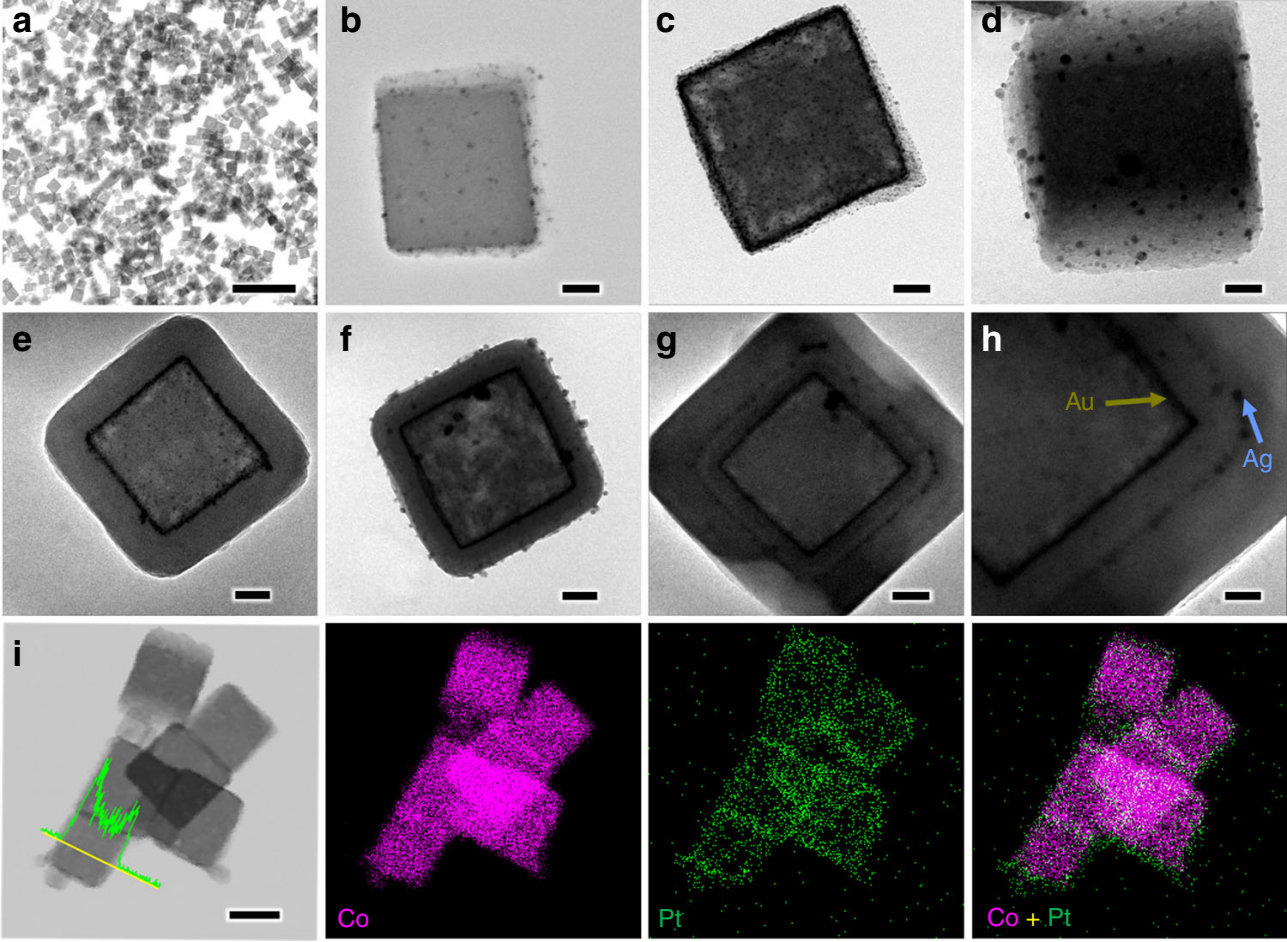
Characterizations of ZIF/metal nanocomposites. **a**, **b** TEM images of ZIF-67/Pt, **c** TEM image of ZIF-67/Au, **d** TEM image of ZIF-67/Ag, **e** TEM image of ZIF-67/Au@ZIF-8, **f** TEM image of ZIF-67/Au@ZIF-8/Ag, **g**, **h** TEM image of ZIF-67/Au@ZIF-8/Ag@ZIF-67, and **i** EDX elemental maps and line scans of ZIF-67/Pt. Colour codes: green represents Pt; pink represents Co. More characterization results of the ZIF/metal nanocomposites can be found in Supplementary Figs. 16-20. Scale bars in **a**–**i** are 1000, 30, 30, 30, 60, 80, 100, 50 and 100 nm, respectively

Subsequently, noble metal nanoparticles were immobilized on the above prepared ZIFs by an in-situ reduction method, in which tetrabutylammonium borohydride (R-NBH_4_) was used as a reducing agent in order to avoid the degradation of ZIFs structure,^23,27^ and HAuCl_4_, H_2_PtCl_6_ and AgNO_3_ methanolic solutions were used as precursor solutions for loading Au, Pt and Ag nanoparticles, respectively. Since the ZIFs have a small theoretical accessible window aperture size of 3.4 Å,^28^ both the metal precursor and R-NBH_4_ cannot diffuse into the cavities which resulted in metal nanoparticles being deposited absolutely on the external surface (Fig. 5a–d). The spatial distribution of the loaded nanoparticles on ZIFs was examined by EDX elemental maps and line scans. As shown in Fig. 5i, it clearly indicates that Pt element is located on the six faces of a nanocube. Furthermore, we find that the alignment between the different ZIFs layers is not affected by the encapsulated metal nanoparticles. It means that the epitaxial growth of a new ZIFs phase on the ZIFs/metal hybrid nanocube could still be realized. Along the same line, a series of very complex ZIFs/metal nanocomposites was successfully produced, such as ZIF-67@ZIF-8/Au@ZIF-67, ZIF-67@ZIF-8/Au@ZIF-67@ZIF-8, ZIF-67/Au@ZIF-8, ZIF-67/Au@ZIF-8/Ag and ZIF-67/Au@ZIF-8/Ag@ZIF-67 (Fig. 5e–h and Supplementary Figs. 16–20). Accordingly, the spatial localization of metal nanoparticles (Au size of 2.8 nm, Pt size of 3 nm or Ag size of 9.4 nm) can be either on the external surface or sandwiched between two ZIFs layers.

### Hydrogen spillover phenomenon

**Fig. 6:**
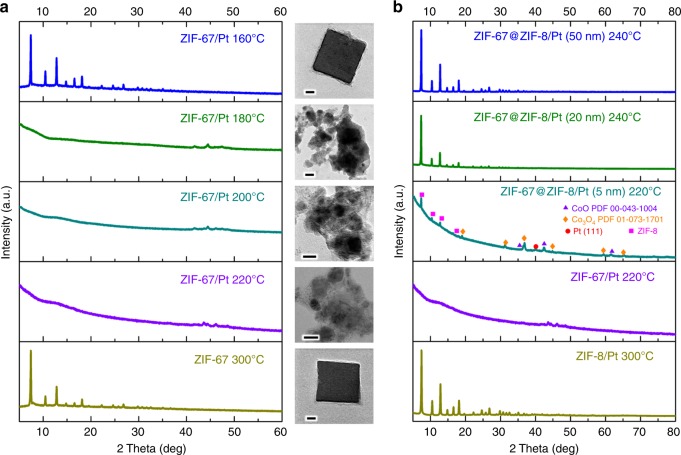
Characterizations of ZIF nanocomposites after soaking in H_2_ at different temperatures. **a** XRD patterns and the corresponding TEM images of various ZIF-67 and ZIF-67/Pt after soaking with flowing H_2_ (4 h), and **b** XRD patterns of ZIF-8/Pt, ZIF-67/Pt and ZIF-67@ZIF-8/Pt nanocomposites with various thickness of ZIF-8 layer under flowing H_2_ (4 h). The structural configurations of the above samples can be referred to Fig. 5 and Supplementary Fig. 27. Scale bars in the inset TEM images from top to bottom are 30, 40, 20, 30 and 30 nm, respectively

**Fig. 7:**
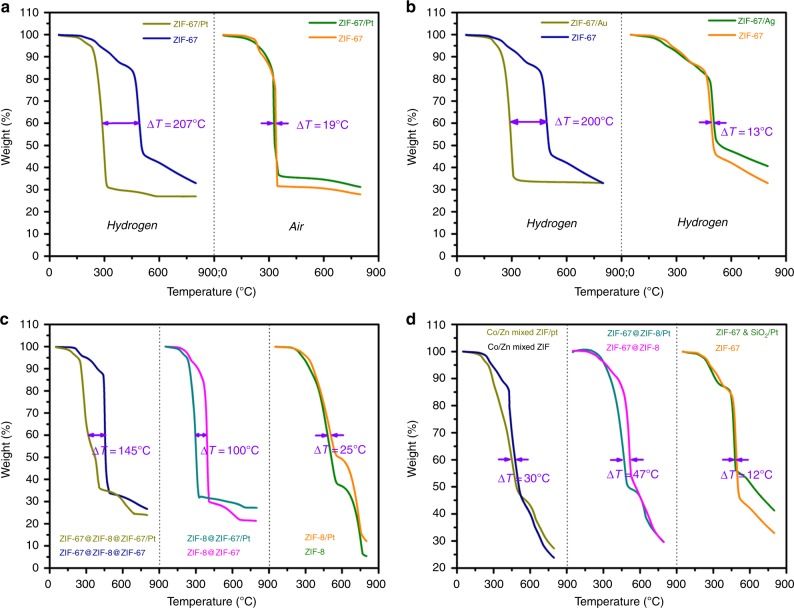
Comparisons of *T*_w60_ in different ZIFs and composites. **a** ZIF-67 with or without Pt loading in different atmospheres, **b** ZIF-67 loading with different metals under flowing H_2_ (H_2_% = 4.2% in N_2_), **c** ZIF-67@ZIF-8@ZIF-67, ZIF-8@ZIF-67 and ZIF-8 with or without Pt loading under flowing H_2_ (H_2_% = 4.2% in N_2_), and **d** Co/Zn mixed ZIF and ZIF-67@ZIF-8 with or without Pt loading and ZIF-67/(SiO_2_/Pt) mixture under flowing H_2_ (H_2_% = 4.2% in N_2_). The structural configurations of the above samples can be referred to Fig. 5

Having thoroughly characterized Matryoshka-type ZIFs/metal nanocubes, we are now in a position to address the issue of hydrogen spillover. We first examined the thermal stabilities of ZIF-67 and ZIF-67/Pt in flowing H_2_ by thermogravimetric analysis (TGA), TEM, and XRD. In the TEM images and XRD patterns in Fig. 6a, indeed, we find that that ZIF-67/Pt was more readily to be decomposed than the pristine ZIF-67. For instance, the ZIF-67 sample could maintain the cubic morphology and structural integrity with a temperature up to 300 °C (temperature lasting for 4 h). However, after loaded with Pt, the onset temperature for ZIF-67 destruction was dropped to 180 °C (at this temperature for 4 h). In the TGA profiles of Fig. 7a, temperatures at which the weight fraction *w* reaches 60 wt% (*T*_w60_) for ZIF-67 and ZIF-67/Pt in H_2_ flows were 493 °C and 286 °C, respectively. The distinct difference of Δ*T*_w60_ (207 °C) must be interpreted by different mechanisms of ZIF-67 decomposition. In the ZIF-67/Pt system, hydrogen atoms were produced by dissociative adsorption of gaseous H_2_ molecules on Pt surface, which subsequently migrated from the Pt to the ZIF-67 by hydrogen spillover. As the split hydrogen atoms have higher reactivity than H_2_, hydrogenolysis of ZIF-67 happened more vigorously, where cobalt–nitrogen single bonds were cleaved and the evolved gases were detected by a mass spectrometer (e.g., NH_3_ and CH_4_, etc., Supplementary Fig. 21). The reduction that occurred to the divalent cobalt ions was further proved by XRD (Supplementary Fig. 22) and XPS (Supplementary Fig. 23). The derived product was also decorated with nitrogen and carbon as proven from the EDX elemental maps (Supplementary Fig. 24). It was found that smaller Pt sizes are more favourable for the fast spillover of the dissociated H onto and subsequent diffusion over the support,^29^ since the smaller Pt can result in higher specific surface areas, more active sites, and greater contacts between the metal nanoparticles and the support (H receptor). In the present work, Pt nanoparticles with an average size of 3 nm were adopted to investigate the hydrogen spillover process.

**Fig. 8:**
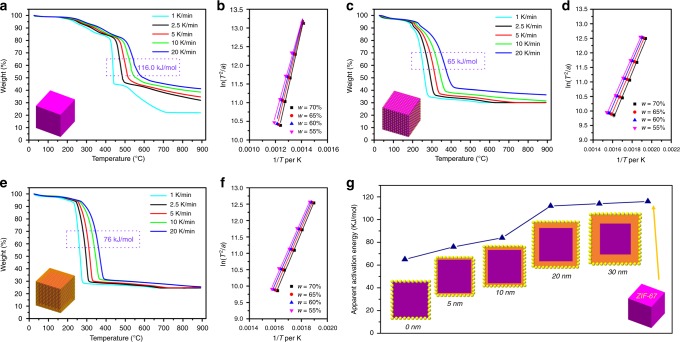
Comparison of the apparent activation energy (*E*_a_) for ZIF-67 hydrogenolysis. **a**, **c**, **e** TGA curves of ZIF-67, ZIF-67/Pt and ZIF-67@ZIF-8/Pt at varying heating rates (1, 2.5, 5, 10 and 20 K min^−1^) in H_2_ flow (100 mL min^−1^, H_2_% = 4.2% in N_2_), **b**, **d**, **f** the corresponding plots of ln(*T*^2^/*ϕ*) versus 1/*T* for the for indicated fractional weight (*w*) of ZIF-67 decomposition, and **g** the comparison of *E*_a_ for ZIF-67 hydrogenolysis in ZIF-67@ZIF-8/Pt with different ZIF-8 layer thickness. The structural configuration of ZIF-67/Pt is shown in Fig. 5a, b, and the structural configurations of ZIF-67@ZIF-8 with different shell thickness are depicted in Supplementary Fig. 27

The activation energies (*E*_a_) of the hydrogenolysis of ZIF-67 by H atoms and H_2_ molecules were also determined by thermogravimetry. The values of *E*_a_ can be calculated from the slope of the plots of ln(*T*^2^/*ϕ*) versus 1/*T* on the same weight fractional *w* based on Eq. (1).^30^

1 $$\ln \left( {\frac{{T_1^2}}{{\emptyset _1}}} \right) - \ln \left( {\frac{{T_2^2}}{{\emptyset _2}}} \right) = \frac{{E_a}}{R}\left( {\frac{1}{{T_1}} - \frac{1}{{T_2}}} \right)$$

wherein, *T*, *R* and *ϕ* are the temperature in Kelvin, the molar gas constant and heating rate, respectively. Figure 8a–d displays the TGA curves under different heating rates and the corresponding plots based on Eq. (1). Accordingly, *E*_a_ values calculated from the slopes are 116 kJ/mol and 65 kJ/mol for the hydrogenolysis of ZIF-67 by H_2_ molecules and H atoms, respectively, which further provides a piece of unambiguous evidence of the hydrogen spillover from Pt to ZIF-67 framework.

In comparison, the thermal stabilities of ZIF-67 and ZIF-67/Pt in an oxidative atmosphere (e.g., air flows) exhibit inappreciable difference (Δ*T*_w60 = _19 ^o^C, Fig. 7a). In addition to ZIF-67/Pt, a promoting effect of Au on ZIF-67 decomposition was also observed (Fig. 7b), as H_2_ dissociate and subsequent diffuse on the supported Au nanoparticles.^31^ In contrast, there was no significant difference between ZIF-67/Ag and ZIF-67 from the TGA profiles (Δ*T*_w60 _= 13 °C, Fig. 7b), revealing that Ag could not dissociate H_2_ under the measuring conditions. A previous study indicates that silica support has poor ability for hydrogen atoms migration.^32^ Therefore, we found that the TGA profile of the mixture of ZIF-67 and SiO_2_/Pt (the mass ratio of Pt to ZIF-67 was kept as 3:97) was almost similar to the pristine ZIF-67 (Δ*T*_w60_ = 12 °C, Fig. 7d), even though hydrogen atoms were produced on Pt surface. Additionally, hydrogen consumption of ZIF-67 and ZIF-67/M (M = metal nanoparticles) samples were monitored by temperature-programmed reduction (TPR). As displayed in Supplementary Fig. 25, it was found that TPR peaks were shifted to a much lower temperature (240 °C) by addition of Pt and Au, as compared with bare ZIF-67 which occurred at 440 °C. The distinct TPR peak could be assigned to ZIF-67 hydrogenolysis (Co^2+^ → Co^0^ transition) as the temperatures of the reduction peaks were well identical to those of maximum weight loss in TGA profiles. However, no significant change was found in the case of ZIF-67/Ag.

In addition, significant deterioration of ZIF-67 due to hydrogen spillover was also found in the samples of ZIF-67@ZIF-8@ZIF-67/Pt and ZIF-8@ZIF-67/Pt (Δ*T*_w60_ were 145 °C and 100 °C, respectively, Fig. 7c). It implies that intimate contact between Pt and ZIF-67 facilitates the migration of hydrogen atoms, thereby enhancing the hydrogenolysis of ZIF-67. However, as also revealed in Fig. 7c, such hydrogen spillover enhanced decomposition was not found in the ZIF-8/Pt system. TEM and XRD data suggest that ZIF-8 phase remains intact in both ZIF-8 and ZIF-8/Pt samples under flowing H_2_ at 300 °C for 4 h (Supplementary Fig. 26). For this reason, no significant H_2_ consumption was found in the corresponding H_2_-TPR profiles of ZIF-8 and ZIF-8/Pt samples (Supplementary Fig. 25). Nonetheless, it could not exclude the transfer of hydrogen atoms in the nonreducible ZIF-8. A control experiment by using Zn/Co mixed ZIF and its supported Pt sample showed that the enhanced effect of ZIF-67 decomposition became not so pronounced (Δ*T*_w60_ = 30 °C, Fig. 7d), which is caused by a lower H migration rate in the presence of zinc ions (Note: Zn^2+^ ions possess fully occupied *d*-orbitals (*d* ^10^), while Co^2+^ ions have unsaturated *d*-orbital (*d* ^7^)). This is consistent with a previous report showing that ZnO was not as effective as TiO_2_ at facilitating hydrogen spillover due to its fully filled *d*-orbital.^32^ Subsequently, we encapsulated ZIF-67 nanocubes with a ZIF-8 shell to study the hydrogen spillover phenomena. Interestingly, TGA data displayed in Fig. 7d show that a small Δ*T*_w60_ (47 °C) of ZIF-67@ZIF-8 and ZIF-67@ZIF-8/Pt was observed as the thickness of ZIF-8 layer was 20 nm. XRD patterns (Fig. 6b) confirm that ZIF-67@ZIF-8/Pt composite with ZIF-8 shell thickness of 20 nm or 50 nm can sustain their structural integrity under flowing H_2_ at 240 °C for 4 h, indicating that almost no hydrogen atom could access to the ZIF-67 core. However, as the ZIF-8 shell was as thin as 5 nm, the intrusion of atomic hydrogen through ZIF-8 layer was observed even at 220 °C for 4 h, leading the hydrogenolysis of ZIF-67. Our XRD investigation further proved that ZIF-8 phase was unchanged, but ZIF-67 phase was severely destroyed and converted to metallic cobalt. It should be noted that cobalt oxide species were found due to the further oxidation of the derived metallic cobalt during sample characterizations (refer to Supplementary Figs. 22, 23).

As depicted in Fig. 1, it is evident that decomposition rate of ZIF-67 is dominated by the concentration of accessed hydrogen atom in the interface of ZIF-67 and ZIF-8 (*C*_*L*_), where the penetration depth (*x*) is equal to the thickness of ZIF-8 shell (*L*). As known, *C*_*x*_ is dominated by the macroscopic diffusion coefficient (*D*_H_), the distance (*x*), and the boundary concentration (*C*_*s*_). According to the random walk model for diffusion, *D*_H_ can be expressed as $D_{\mathrm{H}} = \frac{{va^2}}{{\mathrm{z}}}$, where, *a* is the distance between two neighbouring adsorption sites of hydrogen atom, and *ν* is the microscopic jump frequency, and *z* is the number of adjacent sites to which H-adatoms can hop.^3^ Since, the Pt nanoparticles were all located on {100} facets of ZIFs, so the region is a hydrogen atom rich surface. The diffusion of hydrogen atom can be assumed as one-dimensional diffusion (that is, perpendicular to the surface; Fig. 1). Therefore, in this case *z* = 2. The *ν* depends on temperature in accordance to the Arrhenius expression: $\nu = \nu _0{\mathrm{exp}}\left( {\frac{{ - E_a^ \ast }}{{k_{\mathrm{B}}T}}} \right)$, where *E*_a_^*^ is the activation energy of diffusion, and *ν*_0_ is the “attempt frequency” to jump, *T* is temperature and *k*_B_ is the Boltzmann constant. In this way, three crucial factors would determine the *C*_*x*_ over our model catalysts: hydrogen partial pressure (or concentration), temperature, and thickness of ZIF-8 shell. There are two different endings for the hydrogen atoms migrating in MOFs: one is diffusing atomic H through the ZIF-8 matrix (i.e., described by *C*_*x*_), and the other is Eley-Rideal recombination to form molecular H_2_ which then desorbs as a “free H_2_”. Eley-Rideal recombination step would become predominant as the highly reactive H travelling more deeply into the ZIF-8 shell, leading to the concentration attenuation of H atom.

**Fig. 9:**
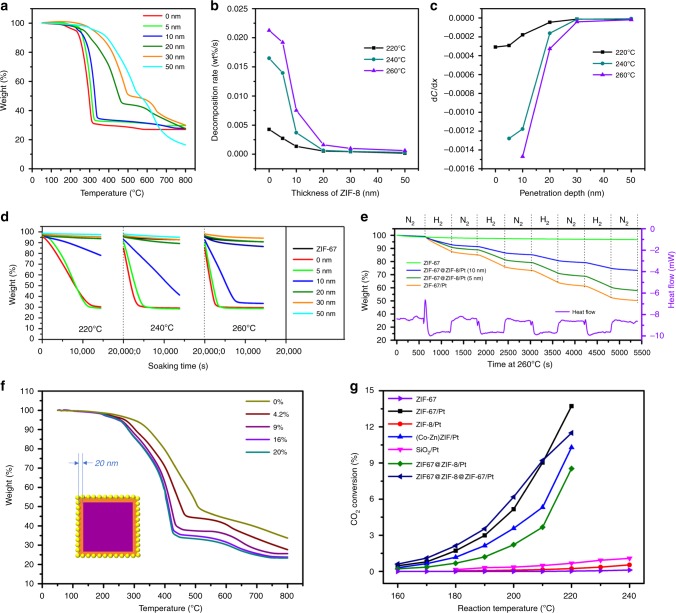
Hydrogen spillover on different ZIF composite materials. **a** TGA profiles of ZIF-67@ZIF-8/Pt with various thickness of ZIF-8 layer under flowing H_2_ (H_2_% = 4.2% in N_2_) with a heating rate of 3 °C min^−1^, **b** the ZIF-8 shell thickness-dependent ZIF-67  decomposition rate, **c** the penetration depth-dependent H concentration gradient (that is, 1st derivative of decomposition rate of ZIF-67) at three different investigated temperatures, **d** time evolution of weight loss of various samples at 220 °C, 240 °C and 260 °C for 4 h under flowing H_2_ (H_2_% = 4.2% in N_2_), **e** TGA profiles of different ZIF composites soaking under five N_2_/H_2_ gas cycles, **f** TGA profiles of ZIF-67@ZIF-8/Pt (ZIF-8 shell thickness of 20 nm) under flowing H_2_ with different concentrations, and **g** catalytic performance of different ZIFs or the composite materials for CO_2_ hydrogenation; reaction conditions: gas flow rate of 12 mL min^−1^, pressure of 30 bar, and solid sample amount of 0.2 g

To further investigate the penetration depth of atomic hydrogen through the ZIF-8 shell. The thickness of ZIF-8 shell could be adjusted as 5 nm, 10 nm, 20 nm, 30 nm and 50 nm by tuning the synthetic parameters. EDX line scanning was used to determine the average thickness of the ZIF-8 coating shell on the ZIF-67 core (as shown in Supplementary Fig. 27). For each catalyst, the Pt content was adjusted in order to maintain a similar surface density (see Method Section). TGA profiles under flowing H_2_ of various ZIF-67@ZIF-8/Pt with a different coating thickness of ZIF-8 are shown in Fig. 9a, and the weight loss of the corresponding samples soaking at a specific temperature (220, 240 and 260 °C for 4 h) are plotted in Fig. 9d. Apparently, the decomposition rate is dependent on both the ZIF-8 thickness and soaking temperature. For instance, the times for full decomposition of ZIF-67 phase in ZIF-67/Pt and ZIF-67@ZIF-8/Pt (10 nm thickness) are 1.4 h and 2.8 h, respectively, under flowing H_2_ at 260 °C. In comparison, even with prolonged time to 18 h, no significant weight loss was found for ZIF-67@ZIF-8/Pt (30 nm thickness) sample under similar conditions (Supplementary Fig. 28). The shell thickness-dependent decomposition rate of ZIF-67 is summarised in Fig. 9b, suggesting that a concentration gradient of accessible atomic hydrogen exists along the penetration depth (see the illustration in Fig. 1d), which is the driving force in spillover process. Essentially, the kinetic energy of H atom flux is caused by the dissociation of the H–H bond. Concentration attenuation of hydrogen atom is attributed to the fact that Eley-Rideal recombination of hydrogen atoms yields H_2_ as travelling through the ZIF-8 shell. As a result, at the same temperature, the degradation rate of ZIF-67 decreases monotonically with increasing the thickness of ZIF-8 shell. Particularly, as illustrated in Fig. 9e, the five N_2_–H_2_–N_2_ cycles during samples soaking at 260 °C further corroborate that the decomposition of ZIF-67 is caused by hydrogen spillover, which also confirms that ZIF-67 hydrogenolysis is an endothermic process. As the steady state was reached, according to Fick’s first law,^33^ the net diffusion flux along the direction *x* is proportional to the concentration gradient (1^st^ derivative): $J = - D_{\mathrm{H}}\frac{{\mathrm{d}}C}{{\mathrm{d}}x}$. The calculated concentration gradient is shown in Fig. 9c, verifying that *J* strikingly decreases along the penetration depth, which thereby weakens the driven force for hydrogen atom diffusion along the penetration. Furthermore, as shown in Fig. 8e–g, *E*_*a*_ values of ZIF-67 hydrogenolysis in ZIF-67@ZIF-8/Pt composites are 76, 84, 112 and 114 kJ/mol, when the ZIF-8 shells are 5, 10, 20 and 30 nm, respectively. The increasing *E*_*a*_ with thicker ZIF-8 implies that the transportation and the population of atomic H depend on the thickness of ZIF-8. This result also suggests that the H migration distance in ZIFs at low temperature (or ambient temperature) will be short if an extrapolation from the above high temperature data is made.

As mentioned above, hydrogen spillover through MOFs in the gas phase is excluded. Therefore, it is deduced that the resultant H atoms were chemisorbed on ZIFs during hydrogen spillover. Because both MeIm ligand (which is deprotonated from H-MeIM) and ZIFs have been widely reported as N-heterocyclic proton carriers via sharing protons on N–H bonds,^34–36^ we believe that such N–H bonds are also present for the diffusion of H atoms through ZIF-8. The process described in Supplementary Fig. 29 can be further considered in future, following the reported work of a combined electron-proton mobility on reducible TiO_2_ support which has an energy barrier *E*_act_ = 0.65 eV and thus is energetically more favourable than other possible diffusion mechanisms (such as oxygen vacancies).^3^ As discussed earlier, a hydrogen atom would also combine with another hydrogen atom to generate H_2_ again (viz., H_gas_ + H_ad_ → H_2,gas_); longer travelling times are spent and thus more recombination events take place along a diffusion path of H atoms. As a result, the concentration of atomic H decreases as the penetration depth increases (Fig. 1e and Supplementary Fig. 30). In addition, since the migration is usually a highly endothermic process, the migration rate of H atoms speeds up pronouncedly at elevated temperatures, which is in good agreement with the decomposition rate of ZIF-67 that increases monotonically with temperature. Again, the above experimental results suggest that the “hydrogen spillover” in ZIF materials at ambient temperature may not contribute significantly to the enhanced hydrogen storage.

In addition, the effect of hydrogen concentration on hydrogen spillover rate was studied by varying the concentration in H_2_/N_2_ mixture (at ambient pressure). Taking ZIF-67@ZIF-8/Pt (shell: 20 nm) as an example (Fig. 9f), increasing hydrogen concentration leads to a higher ZIF-67 decomposition rate. This is due to the change of boundary concentration (*C*_*s*_) of atomic hydrogen at the gas-Pt interface, where higher hydrogen gas concentration gave rise to a higher *C*_*s*_ (see Fig. 1d).^37^ However, almost identical TGA profiles were found at hydrogen concentrations of 16% and 20%, due to reaching the equilibrium condition. Furthermore, hydrogen spillover occurring at high pressure (30 bar) was also investigated at temperatures between 160–240 °C. Since TGA apparatus cannot be conducted under high pressure, a fixed bed gas-phase CO_2_ hydrogenation reaction was utilized as a probe for ZIF-67 decomposition due to hydrogen spillover at high pressure (30 bar, Fig. 9g). In such a case, Pt nanoparticles were able to dissociate H_2_ molecule which then migrated as H atoms through the ZIF-8 shell to reduce the cobalt ions in the ZIF-67 core. In this process, the activity of CO_2_ hydrogenation can be interpreted as originating from the metallic Co derived from hydrogenolysis of ZIF-67, since cobalt is an active centre for CO_2_ hydrogenation towards methane and CO.^38,39^ In accordance with the above information, the ZIF-8 phase in ZIF-8/Pt sample was quite stable even in a harsh operating condition (240 °C, 30 bar, H_2_% = 72%). Likewise, in the absence of Pt, the pristine ZIF-67 was not altered after the reaction (30 bar, 240 °C, 12 h, refer to XRD pattern in Supplementary Fig. 31), showing negligible CO_2_ conversion over the entire temperature range, similar to the cases of ZIF-8/Pt and SiO_2_/Pt. On the contrary, ZIF-67/Pt totally converted to Co/Pt bimetallic catalysts, which showed the highest CO_2_ conversion. A positive correlation was found between the CO_2_ conversion and the hydrogen spillover rate. Notably, if the ZIF-67 phase has a close contact with Pt (e.g., ZIF-67/Pt and ZIF-67@ZIF-8@ZIF-67/Pt), the catalysts exhibited higher activities. Obviously, the introduction of ZIF-8 layer between ZIF-67 and Pt, retards the hydrogenolysis rate of ZIF-67, thereby decreasing the CO_2_ hydrogenation activity at any measured temperatures. Although hydrogen atoms cannot reduce the ZIF-67@ZIF-8/Pt (shell: 20 nm) at normal atmospheric pressure, it was observed that the ZIF-67 phase in the sample was totally decomposed at 30 bar, suggesting that a higher amount of available hydrogen atoms could migrate to ZIF-67 core at higher pressure. XRD patterns and TEM images of the spent catalyst samples were analysed in Supplementary Fig. 32 to confirm ZIF structures transformation during the reaction.

## Discussion

We have demonstrated an ingenious way to prove the spillover of hydrogen atoms in ZIF-8 layer at elevated temperatures, by using a variety of well-defined hybrid ZIFs/Pt nanocubes as probes. Firstly, a simple approach has been developed to fabricate multi-shell structured ZIFs with Matryoshka doll-like features via step-by-step heteroepitaxial growth in solution. For core–shell structured ZIF-67@ZIF-8/Pt, the enhanced decomposition of ZIF-67 at low temperatures could be attributed to the hydrogen spillover through the ZIF-8 shell (where molecular H_2_ is nonreactive). On the other hand, the hydrogenolysis of ZIF-67 provides direct information about hydrogen atoms spillover which exclusively migrates through {100} facets of ZIF-8 crystalline shell. It is evident that the diffusion behaviour of hydrogen atoms is dominated by the temperature, hydrogen concentration and pressure, and shell thickness. In addition, we have proposed a plausible migration mechanism to elucidate long-distance transport of hydrogen atoms over a ZIF-8 layer, although more theoretical work is still needed for the future investigation of atomic H migration in MOFs.

In contrast to hydrogen spillover phenomenon in hydrogenation catalysis, ambiguity exists about the spillover involved in hydrogen storage enhancement, mostly at ambient temperature. Our experimental results not only provide direct evidence for hydrogen spillover in ZIFs at elevated temperatures, but also set up an upper limit of the migration distance for H atoms diffusing in ZIF-8 phase at elevated temperatures. In terms of hydrogen storage, our findings also indicate that hydrogen spillover in ZIF-8 must have a limited spatial scope (i.e., in the vicinity of noble metal particles) at ambient temperature. The key to measuring the diffusion path of H atoms is that the shell of ZIF-8 serves as a spatial ruler while the core of ZIF-67 functions as a hydrogen atom detector through hydrogenolysis. As demonstrated in the present work, future investigations on hydrogen spillover processes in other MOFs can also be carried out by constructing MOFs and noble-metal nanocomposites with the similar Matryoshka configuration.

## Methods

### Materials

The following chemicals were used as received without further purification: zinc nitrate hexahydrate (Aldrich, 98%), cobalt (II) nitrate hexahydrate (Aldrich, 98 + %), 2-methylimidazole (H-MeIm, 99%, Aldrich), cetyltrimethylammonium bromide (CTAB, 98%, Aldrich), chloroplatinic acid hydrate (Sigma-Aldrich, 99.9 + %), gold (III) chloride trihydrate (Sigma, 99.9%), silver nitrate (Merck, 99%), tetrabutylammonium borohydride (R-NBH_4_, 98%, Sigma), sodium hydroxide (Merck, 99%), hydrochloric acid (VWR chemical, 32%), methanol (Fisher, 99.99%), and ethanol (Fisher, 99.99%). Deionized water was used for all experiments.

### Nanostructure fabrications

For the synthesis of ZIF-67, 0.3 mL of Co(NO_3_)_2_ aqueous solution (50 g L^−1^, 0.172 M) was mixed with 2 mL of water and 0.9 mL of H-MeIm aqueous solution (200 g L^−1^, 2.44 M). After stirring for 2 min, 0.2 mL of CTAB aqueous solution (50 g L^−1^) was added. The mixture was kept stirring for 6 h at room temperature. The purple colour product was harvested through centrifugation and washing (with ethanol for three times). Finally, the product was dispersed into 2 mL of ethanol for TEM analysis and further use. The yield of the product based on Co salts was ca. 91%. For the synthesis of ZIF-8, 0.2 mL of Zn(NO_3_)_2_ aqueous solution (50 g L^−1^) was used as the metal source while other parameters were held to identical values as ZIF-67 synthesis. Similarly, in the case of cubic Zn/Co-ZIFs preparation, 0.15 mL of Zn(NO_3_)_2_ aqueous solution (50 g L^−1^) and 0.15 mL of Co(NO_3_)_2_ aqueous solution (50 g L^−1^) were used as the metal source. To construct multi-layered ZIFs (viz., (ZIFs@)_*n*−1_ZIFs), we first prepared ZIFs nanocubes and used them as core crystals in a solution containing metal ions and H-MeIm. Usually, the amount of ZIFs core crystals was 0.5 mL at a concentration of 5 mg mL^−1^, and 0.2 mL of CTAB aqueous solution (50 g L^−1^) was used in each batch. Other chemicals were added in a similar way as the preparation of ZIF-67 or ZIF-8. As for loading Pt nanoparticles on the ZIF nanocubes, an in-situ reduction method was employed.^40^ Briefly, 200 mg of a specific ZIFs was dispersed in 40 mL of methanol by sonication for 10 min, before the addition of 8 mL of H_2_PtCl_6_ methanolic solution (10 mM). The mixture was vigorously stirred for 1 h at room temperature. Then 20 mL of 0.25 M R-NBH_4_ methanolic solution (used around 8 min just after preparation) was injected into the above mixture. After stirring for 30 min, the product was collected through centrifugation and washing procedures (with ethanol twice). The loading amount of Pt was adjusted according to the size of ZIF nanocubes, in order to achieve the same outer surface loading of Pt. For the ZIFs/Pt catalysts (ZIF-67/Pt, ZIF-8/Pt and Zn/Co-ZIF/Pt), the Pt loading amount was maintained as 3 wt%. The immobilization of Au and Ag nanoparticles were prepared in a similar manner but using HAuCl_4_ and AgNO_3_ as the metal precursors.

### Characterization techniques

Sample morphologies of our (ZIFs@)_*n*−1_ZIFs were investigated with transmission electron microscopy (TEM, JEM-2010, 200 kV) and high-resolution TEM (HRTEM, JEM-2100F, 200 kV). Crystallographic information of these samples was established by X-ray diffraction (XRD, Bruker D8 Advance) using Cu *K*_α_ radiation. The compositional analysis of our samples was carried out by energy-dispersive X-ray spectroscopy (EDX, Oxford Instruments, Model 7426). The determinations of specific surface areas, pore volume, and pore size of our samples were made using N_2_ physisorption isotherms at 77 K (Quantachrome NOVA-3000 system). Metal contents in our catalysts were measured by inductively coupled plasma optical emission spectrometry (ICP-OES, Optima 7300DV, Perkin Elmer), and the analysis on surface compositions of the samples was made by X-ray photoelectron spectroscopy (XPS, AXIS-HSi, Kratos Analytical). The binding energies in our XPS spectra were corrected with the reference binding energy of adventitious carbon C 1 s at 284.5 eV. TGA results were obtained by using a Mettler Toledo thermogravimetric analyser at a ramping rate of 3 °C min^−1^ (sample mass ~20 mg on 150 μL aluminium pan). The total gas flowrate was set at 120 mL min^−1^. Temperature-programmed reduction analysis (H_2_-TPR) was carried out by heating a sample (30 mg) in a quartz tube using HIDEN analytical CATLAB instrument. The sample was heated from 50 to 750 °C at 3 °C min^−1^ in a flow of 5 vol% H_2_/Ar mixture (60 mL min^−1^ at a total pressure of 1 atm). The system was stabilized for 10 min at 50 °C to obtain a straight baseline. The amount of hydrogen consumption and evolved gases (e.g., H_2_O, NH_3_, and CH_4_) were monitored by a mass spectrometer (MS).

### Catalytic performance evaluations

The CO_2_ hydrogenation reactions were carried out in a continuous flow fixed bed reactor (3/8 inch stainless steel) loading with 200 mg of ZIF samples. The flowchart of the catalytic experimental set-up is shown in Supplementary Fig. 33. A gas stream with CO_2_/H_2_/N_2_ = 24%/72%/4% (where  N_2_ acted as an internal standard) was fed at a flowrate manipulated by a Brooks mass flow controller. The reactor temperature was monitored with a thermocouple attached to the centre of catalyst bed. The reactor pressure was tuned by a back-pressure gas regulator. Compositions of reactants and products were determined online with a gas chromatography apparatus equipped with FID and TCD detectors.

## Electronic supplementary material

Supplementary Information

## Data Availability

The data supporting the findings of this study are available upon request from the corresponding author (including data presented in the main text and in the Supplementary Information).

## References

[1] Khoobiar S (1964). Particle to particle migration of hydrogen atoms on platinum—alumina catalysts from particle to neighboring particles. J. Phys. Chem..

[2] Prins R (2012). Hydrogen spillover. Facts and fiction. Chem. Rev..

[3] Karim W (2017). Catalyst support effects on hydrogen spillover. Nature.

[4] Im J, Shin H, Jang H, Kim H, Choi M (2014). Maximizing the catalytic function of hydrogen spillover in platinum-encapsulated aluminosilicates with controlled nanostructures. Nat. Commun..

[5] Beaumont SK, Alayoglu S, Specht C, Kruse N, Somorjai GA (2014). A nanoscale demonstration of hydrogen atom spillover and surface diffusion across silica using the kinetics of CO_2_ methanation catalyzed on spatially separate Pt and Co nanoparticles. Nano Lett..

[6] Li Y, Yang RT (2006). Significantly enhanced hydrogen storage in metal−organic frameworks via spillover. J. Am. Chem. Soc..

[7] Li Y, Yang RT (2006). Hydrogen storage in metal−organic frameworks by bridged hydrogen spillover. J. Am. Chem. Soc..

[8] Liu YY, Zeng JL, Zhang J, Xu F, Sun LX (2007). Improved hydrogen storage in the modified metal-organic frameworks by hydrogen spillover effect. Int. J. Hydrog. Energy.

[9] Gutiérrez I, Díaz E, Ordóñez S (2013). Consequences of cavity size and palladium addition on the selective hydrogen adsorption in isoreticular metal-organic frameworks. Thermochim. Acta.

[10] Campesi R, Cuevas F, Latroche M, Hirscher M (2010). Hydrogen spillover measurements of unbridged and bridged metal-organic frameworks-revisited. Phys. Chem. Chem. Phys..

[11] Szilagyi PA (2014). Probing hydrogen spillover in Pd@MIL-101(Cr) with a focus on hydrogen chemisorption. Phys. Chem. Chem. Phys..

[12] He T, Pachfule P, Wu H, Xu Q, Chen P (2016). Hydrogen carriers. Nat. Rev. Mater..

[13] Psofogiannakis GM, Froudakis GE (2011). Fundamental studies and perceptions on the spillover mechanism for hydrogen storage. Chem. Commun..

[14] Psofogiannakis GM, Froudakis GE (2011). Theoretical explanation of hydrogen spillover in metal−organic frameworks. J. Phys. Chem. C..

[15] Lee K (2010). Hole-mediated hydrogen spillover mechanism in metal-organic frameworks. Phys. Rev. Lett..

[16] Luzan SM, Talyzin AV (2010). Hydrogen adsorption in Pt catalyst/MOF-5 materials. Microporous Mesoporous Mater..

[17] Langmi HW, Ren J, North B, Mathe M, Bessarabov D (2014). Hydrogen storage in metal-organic frameworks: a review. Electrochim. Acta.

[18] Broom DP, Hirscher M (2016). Irreproducibility in hydrogen storage material research. Energy Environ. Sci..

[19] Tang J (2015). Thermal conversion of core–shell metal–organic frameworks: a new method for selectively functionalized nanoporous hybrid carbon. J. Am. Chem. Soc..

[20] Chen H, Wang L, Yang J, Yang RT (2013). Investigation on hydrogenation of metal–organic frameworks HKUST-1, MIL-53, and ZIF-8 by hydrogen spillover. J. Phys. Chem. C.

[21] Stuckert NR, Wang L, Yang RT (2010). Characteristics of hydrogen storage by spillover on Pt-doped carbon and catalyst-bridged metal organic framework. Langmuir.

[22] Yin Y (2017). Modification of as synthesized SBA-15 with Pt nanoparticles: nanoconfinement effects give a boost for hydrogen storage at room temperature. Sci. Rep..

[23] Li Z, Zeng HC (2013). Surface and bulk integrations of single-layered Au or Ag nanoparticles onto designated crystal planes {110} or {100} of ZIF-8. Chem. Mater..

[24] Pan Y (2011). Tuning the crystal morphology and size of zeolitic imidazolate framework-8 in aqueous solution by surfactants. CrystEngComm.

[25] Yang J (2015). Hollow Zn/Co ZIF particles derived from core–shell ZIF-67@ZIF-8 as selective catalyst for the semi-hydrogenation of acetylene. Angew. Chem..

[26] Wannapaiboon S (2015). Hierarchical structuring of metal-organic framework thin-films on quartz crystal microbalance (QCM) substrates for selective adsorption applications. J. Mater. Chem. A.

[27] Zhan G, Zeng HC (2016). Integrated nanocatalysts with mesoporous silica/silicate and microporous MOF materials. Coord. Chem. Rev..

[28] Park KS (2006). Exceptional chemical and thermal stability of zeolitic imidazolate frameworks. Proc. Natl Acad. Sci. USA.

[29] Wang L, Stuckert NR, Chen H, Yang RT (2011). Effects of Pt particle size on hydrogen storage on Pt-doped metal−organic framework IRMOF-8. J. Phys. Chem. C.

[30] Nishida H, Yamashita M, Hattori N, Endo T, Tokiwa Y (2000). Thermal decomposition of poly(1,4-dioxan-2-one). Polym. Degrad. Stab..

[31] Panayotov DA, Yates JT (2007). Spectroscopic detection of hydrogen atom spillover from Au nanoparticles supported on TiO_2_: Use of conduction band electrons. J. Phys. Chem. C.

[32] Collins SSE, Cittadini M, Pecharromán C, Martucci A, Mulvaney P (2015). Hydrogen spillover between single gold nanorods and metal oxide supports: a surface plasmon spectroscopy study. ACS Nano.

[33] Waser R (1986). Diffusion of hydrogen defects in BaTiO_3_ ceramics and SrTiO_3_ single crystals. Ber. Bunsenges. Phys. Chem..

[34] Xu H, Tao S, Jiang D (2016). Proton conduction in crystalline and porous covalent organic frameworks. Nat. Mater..

[35] Barbosa P, Rosero-Navarro NC, Shi FN, Figueiredo FML (2015). Protonic conductivity of nanocrystalline zeolitic imidazolate framework 8. Electrochim. Acta.

[36] Sen U (2016). Proton conducting self-assembled metal–organic framework/polyelectrolyte hollow hybrid nanostructures. ACS Appl. Mater. Interfaces.

[37] Liu Y, Li Y, Huang P, Song H, Zhang G (2016). Modeling of hydrogen atom diffusion and response behavior of hydrogen sensors in Pd–Y alloy nanofilm. Sci. Rep..

[38] Jimenez J, Bird A, Santos Santiago M, Wen C, Lauterbach J (2017). Supported cobalt nanorod catalysts for carbon dioxide hydrogenation. Energy Technol..

[39] Zhan G, Zeng HC (2017). ZIF-67-derived nanoreactors for controlling product selectivity in CO_2_ hydrogenation. ACS Catal..

[40] Zhan G, Zeng HC (2017). Smart nanocatalysts with streamline shapes. ACS Cent. Sci..

