# Supplementary material for: Hydrogen spillover through Matryoshka-type (ZIFs@)n−1ZIFs nanocubes
Source: Nat Commun. 2018 Sep 17;9:3778. doi: 10.1038/s41467-018-06269-z (PMC6141604; doi:10.1038/s41467-018-06269-z)
Supplement: Supplementary file 1 — Supplementary Information [file 41467_2018_6269_MOESM1_ESM.pdf]

Supplementary Information

Hydrogen spillover through Matryoshka-type $(ZIFs@)_{n-1}ZIFs$ nanocubes

*Guowu Zhan and Hua Chun Zeng**

Department of Chemical and Biomolecular Engineering, Faculty of Engineering, National University of Singapore, 10 Kent Ridge Crescent, Singapore 119260
Cambridge Centre for Advanced Research in Energy Efficiency in Singapore, 1 Create Way, Singapore 138602, Singapore

*E-mail: chezhc@nus.edu.sg

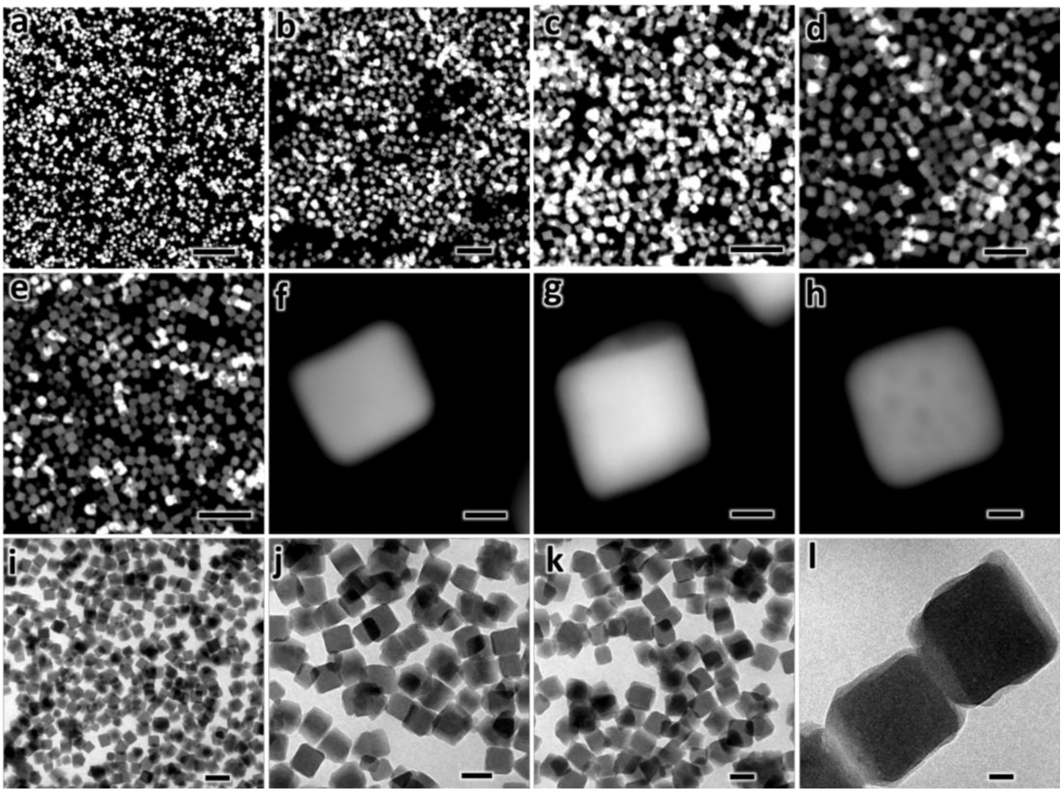

Supplementary Figure 1. (a-h) High-angle annular dark-field scanning TEM (HAADF-STEM) images (at different magnifications) and (i-l) TEM images (at different magnifications) of ZIF-8 nanocubes. Scale bars in (a-l) are 1000 nm, 500 nm, 500 nm, 300 nm, 500 nm, 30 nm, 30 nm, 20 nm, 200 nm, 100 nm, 100 nm, and 20 nm, respectively.

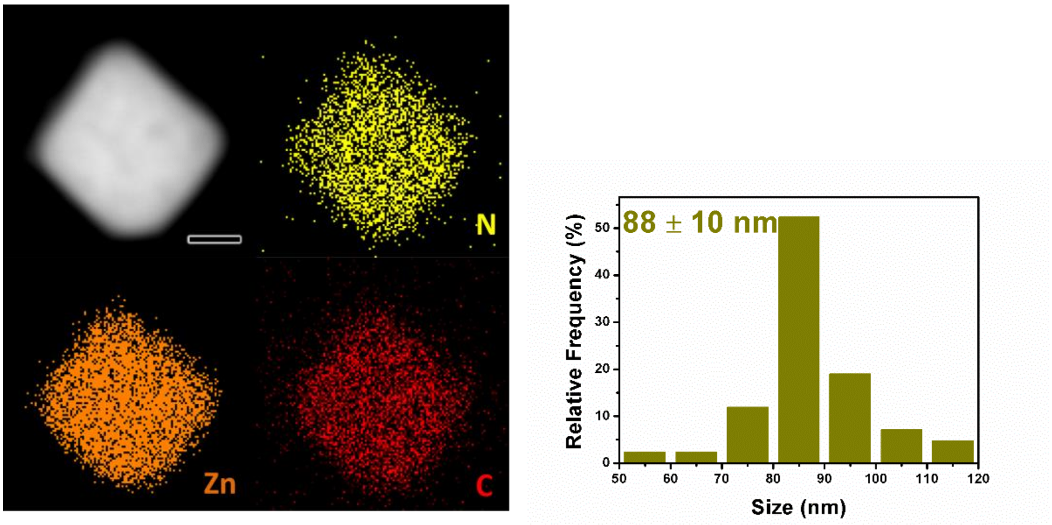

Supplementary Figure 2. EDX elemental maps and statistics of particle size of ZIF-8 nanocubes. Colour codes: red = carbon, yellow = nitrogen, and brown = zinc. Scale bar in the STEM image is 30 nm.

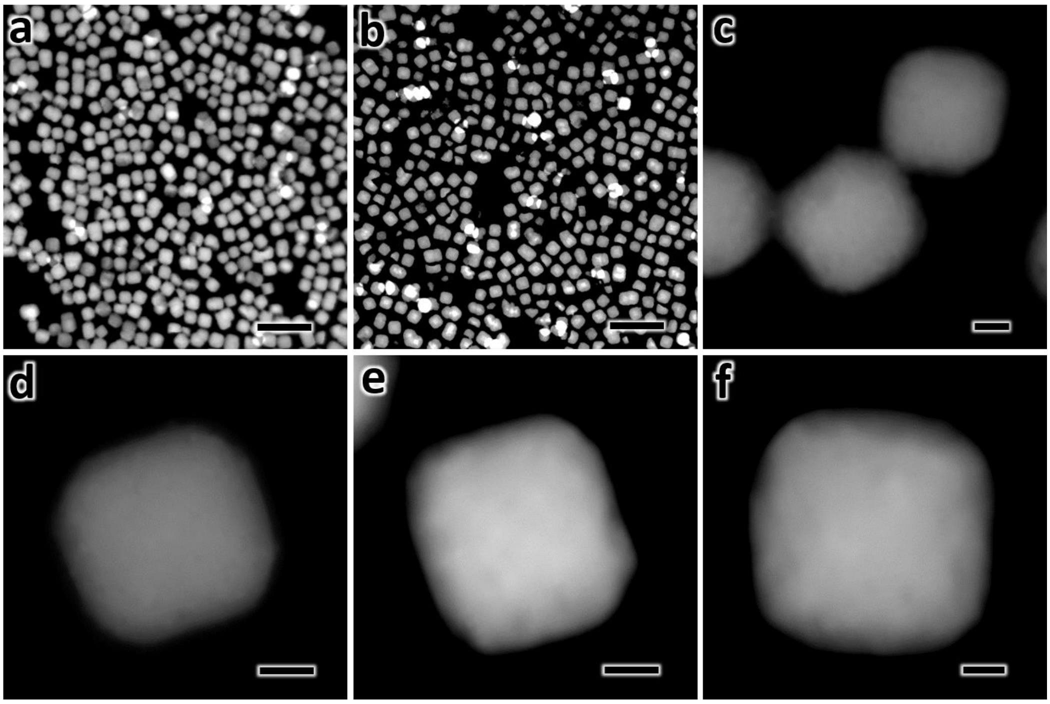

Supplementary Figure 3. (a-f) High-angle annular dark-field scanning TEM (HAADF-STEM) images (at different magnifications) of (ZIFs@)$_{n-1}$ZIFs, when n = 2 (*i.e.*, ZIF-8@ZIF-67). Scale bars in (a-f) are 1000 nm, 1000 nm, 50 nm, 50 nm, 50 nm, and 30 nm, respectively.

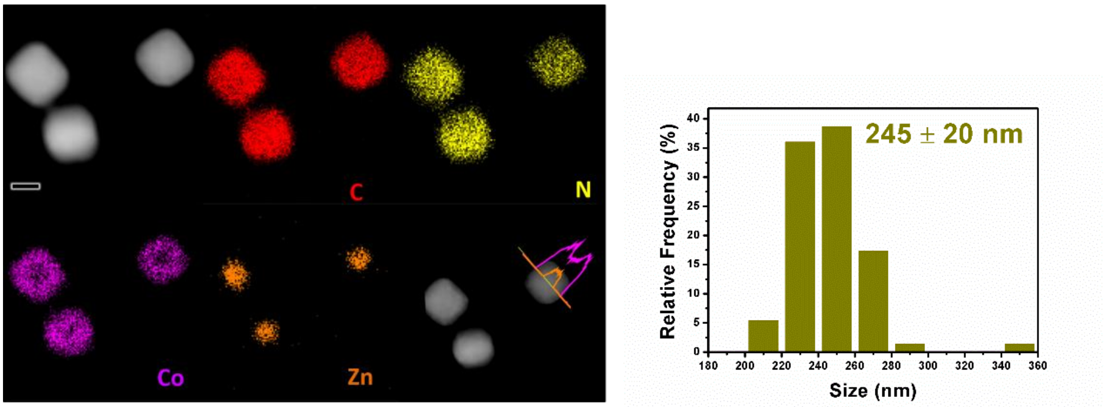

Supplementary Figure 4. EDX elemental maps and statistics of particle size of ZIF-8@ZIF-67. Colour codes: red = carbon, yellow = nitrogen, pink = cobalt, and brown = zinc. Scale bar in the STEM image is 100 nm.

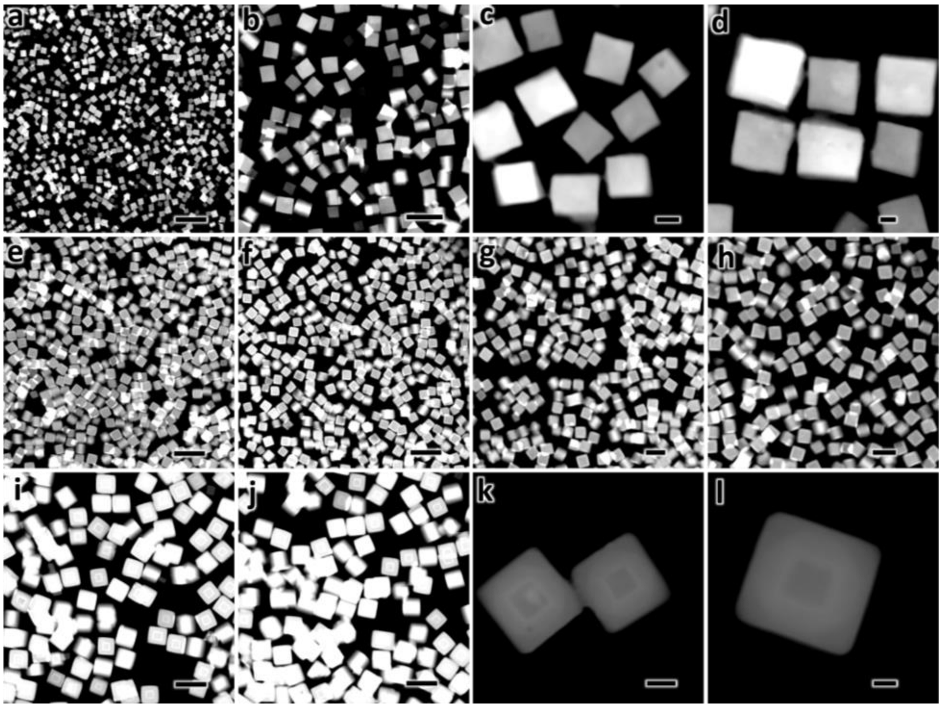

Supplementary Figure 5. High-angle annular dark-field scanning TEM (HAADF-STEM) images (at different magnifications) of ZIF-67 (a-d), ZIF-67@ZIF-8 (e-h), and ZIF-67@ZIF-8@ZIF-67 (i-l). Scale bars in (a-l) are 1000 nm, 500 nm, 100 nm, 50 nm, 1000 nm, 1000 nm, 500 nm, 500 nm, 1000 nm, 1000 nm, 200 nm and 100 nm, respectively.

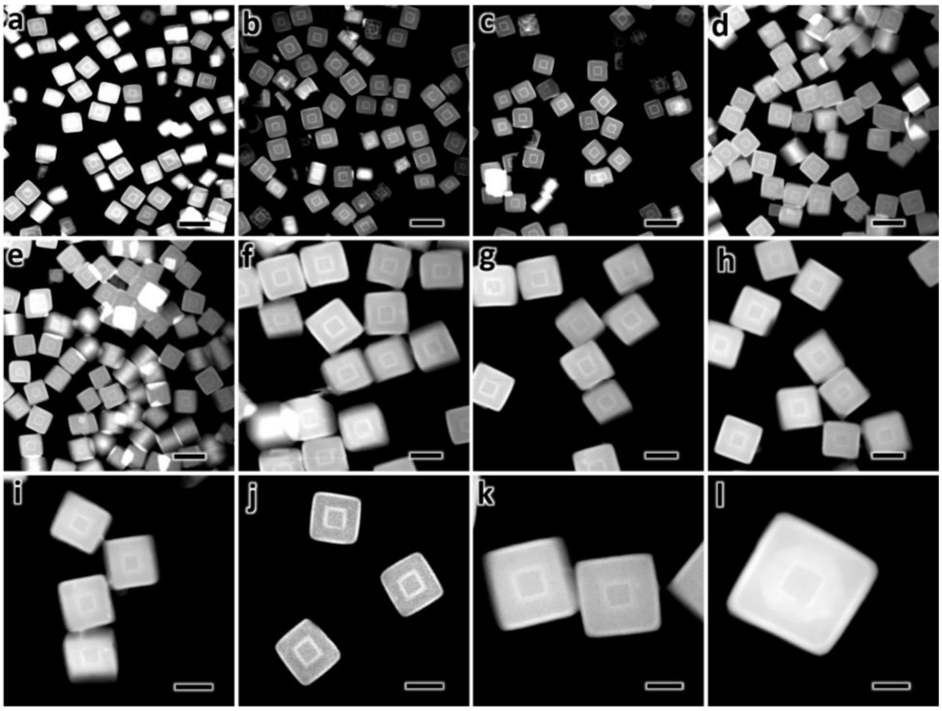

Supplementary Figure 6. High-angle annular dark-field scanning TEM (HAADF-STEM) images (at different magnifications) of tetra-layered ZIFs (i.e., $(ZIFs@)_{n-1}ZIFs$, when n = 4). Scale bars in (a-e) are 1000 nm, scale bars in (f-j) are 500 nm, scale bar in (k) is 300 nm, and scale bar in (l) is 200 nm.

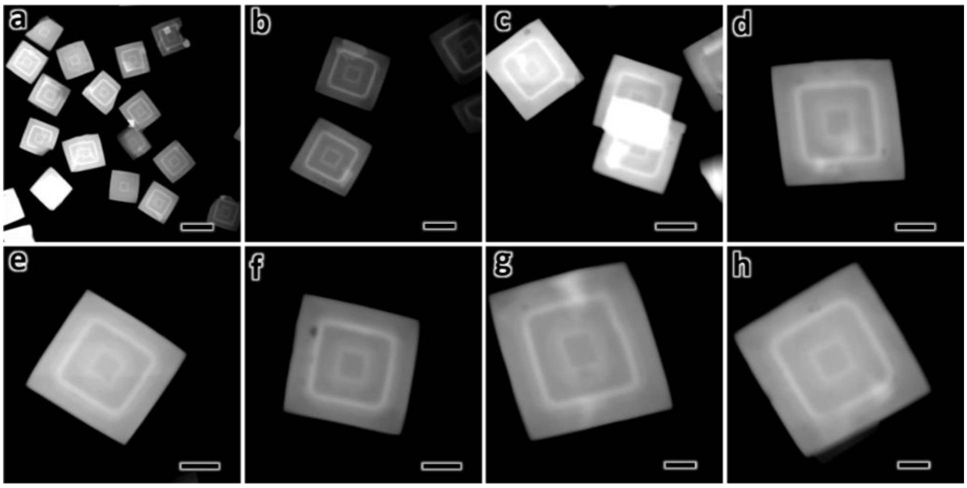

Supplementary Figure 7. (a-h) High-angle annular dark-field scanning TEM (HAADF-STEM) images (at different magnifications) of penta-layered ZIFs (i.e., $(ZIFs@)_{n-1}ZIFs$, when n = 5). Scale bars in (a-h) are 1000 nm, 500 nm, 500 nm, 300 nm, 300 nm, 300 nm, 200 nm, and 200 nm, respectively.

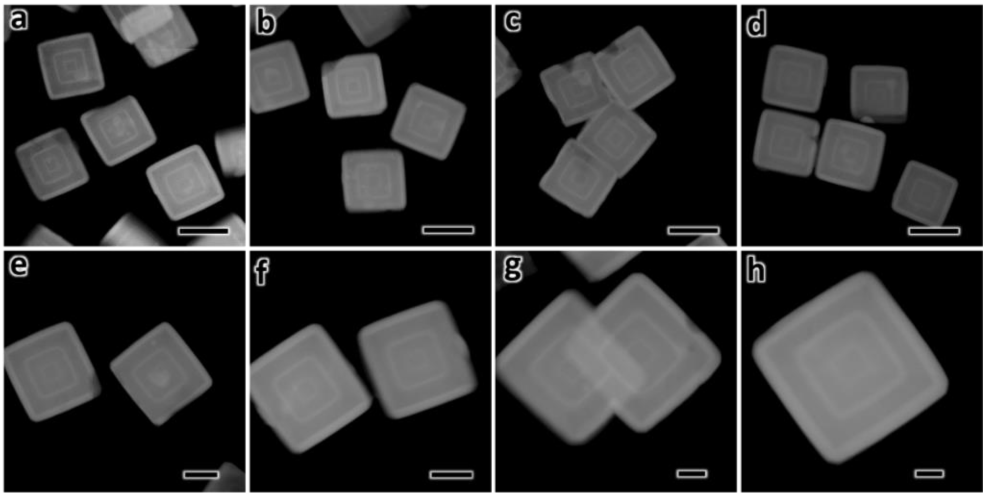

Supplementary Figure 8. (a-h) High-angle annular dark-field scanning TEM (HAADF-STEM) images (at different magnifications) of hexa-layered ZIFs (i.e., $(ZIFs@)_{n-1}ZIFs$, when n = 6). Scale bars in (a-h) are 1000 nm, 1000 nm, 1000 nm, 1000 nm, 500 nm, 500 nm, 300 nm, and 200 nm, respectively.

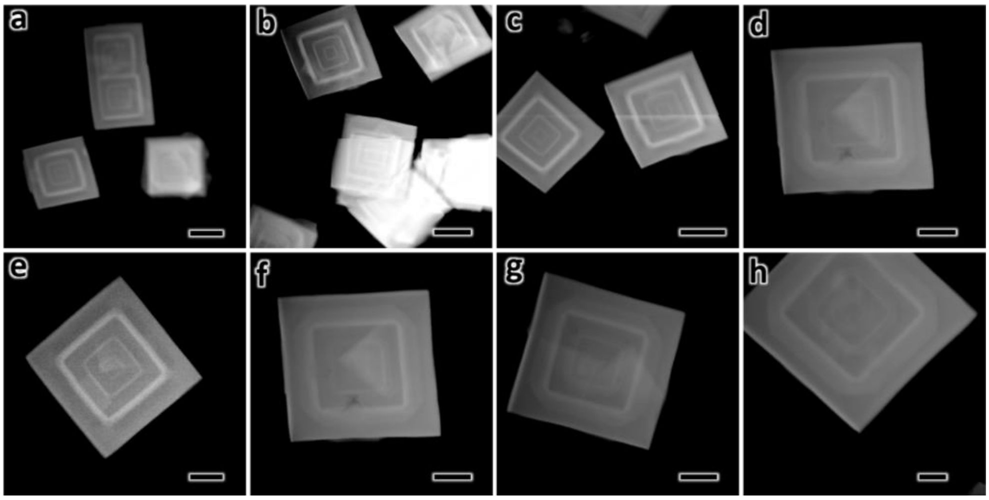

Supplementary Figure 9. (a-h) High-angle annular dark-field scanning TEM (HAADF-STEM) images (at different magnifications) of hepta-layered ZIFs (i.e., $(ZIFs@)_{n-1}ZIFs$, when n = 7). Scale bars in (a-h) are 1000 nm, 1000 nm, 1000 nm, 500 nm, 500 nm, 500 nm, 500 nm, and 300 nm, respectively.

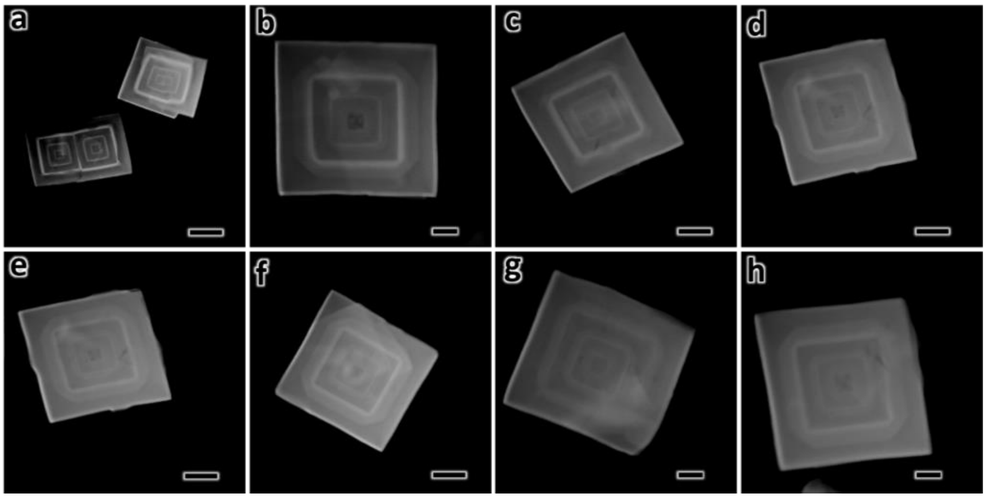

Supplementary Figure 10. (a-h) High-angle annular dark-field scanning TEM (HAADF-STEM) images (at different magnifications) of octa-layered ZIFs (i.e., $(ZIFs@)_{n-1}ZIFs$, when n = 8). Scale bars in (a-h) are 1000 nm, 300 nm, 500 nm, 500 nm, 500 nm, 500 nm, 300 nm, and 300 nm, respectively.

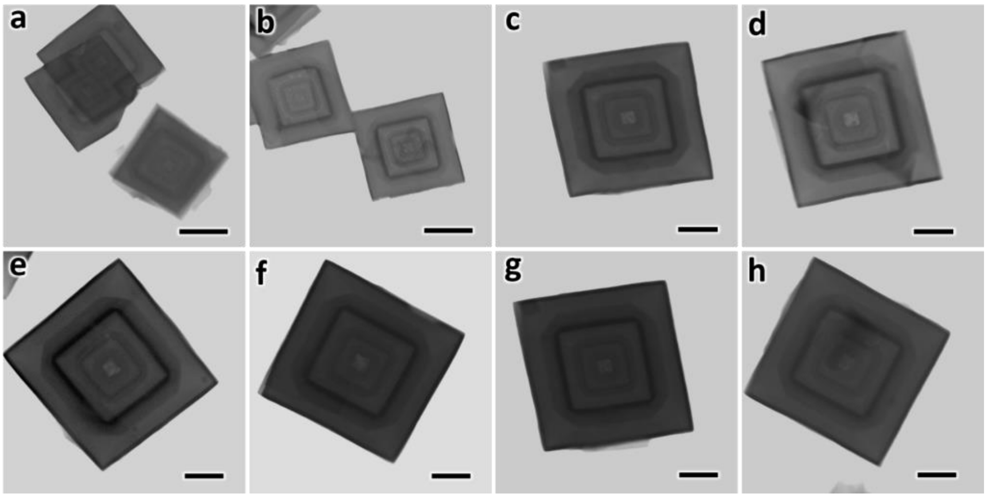

Supplementary Figure 11. (a-h) Typical TEM images (at different magnifications) of octa-layered ZIFs (i.e., (ZIFs@)$_{n-1}$ZIFs, when n = 8). Scale bars in (a, b) are 1000 nm, and scale bars in (c-h) are 500 nm.

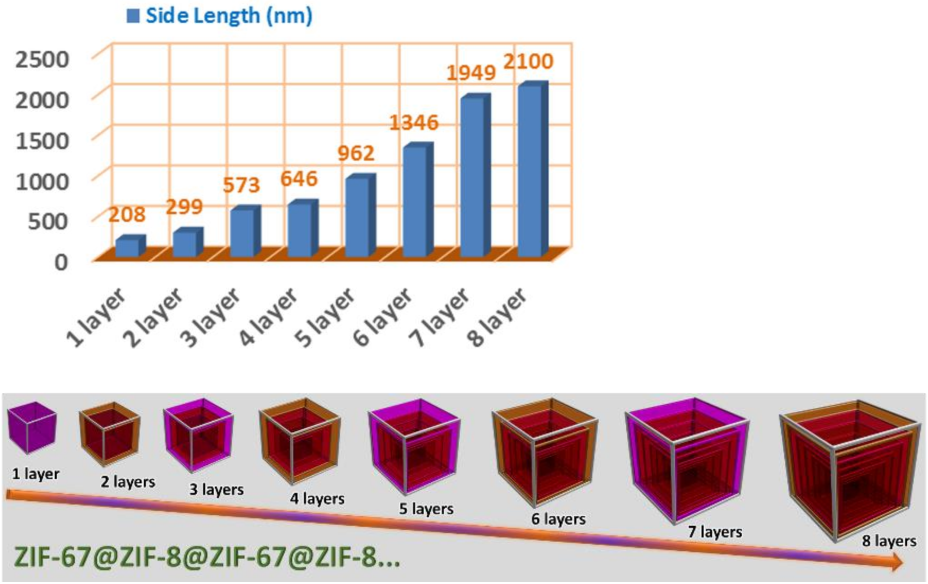

Supplementary Figure 12. Change of side length of Matryoshka-type (ZIFs@)$_{n-1}$ZIFs nanocubes (n = 1 to 8).

Comments: In the above figure, "1 layer" sample represents the starting ZIF-67 core, "2 layers" sample represents ZIF-67@ZIF-8, "3 layers" sample represents ZIF-67@ZIF-8@ZIF-67, and "4 layers" sample represents ZIF-67@ZIF-8@ZIF-67@ZIF-8, and so on.

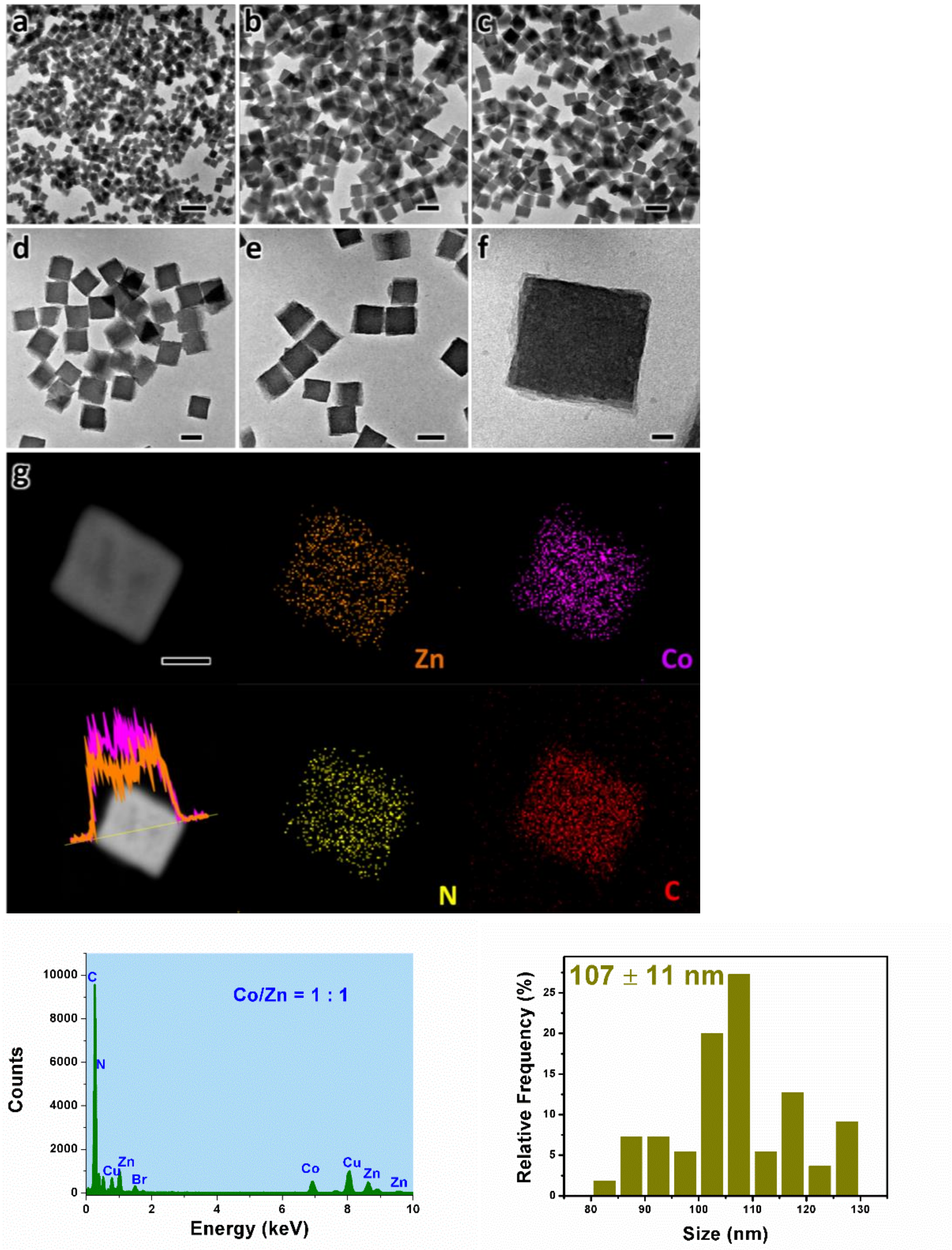

Supplementary Figure 13. Characterizations (TEM images, EDX elemental maps, EDX spectrum, and statistics of particle size) of Zn/Co-ZIF nanocubes. Scale bars in (a-g) are 400 nm, 200 nm, 200 nm, 100 nm, 100 nm, 20 nm, and 50 nm, respectively.

Comments: As shown, Co^{2+} and Zn^{2+} ions are randomly distributed over the metal nodes sites in the ZIF structure, indicating that they are homogeneously mixed. And the average size of Zn/Co-ZIF is 107 nm which is ranged between the size of ZIF-67 nanocubes (208 nm) and ZIF-8 nanocubes (88 nm) prepared under similar conditions.

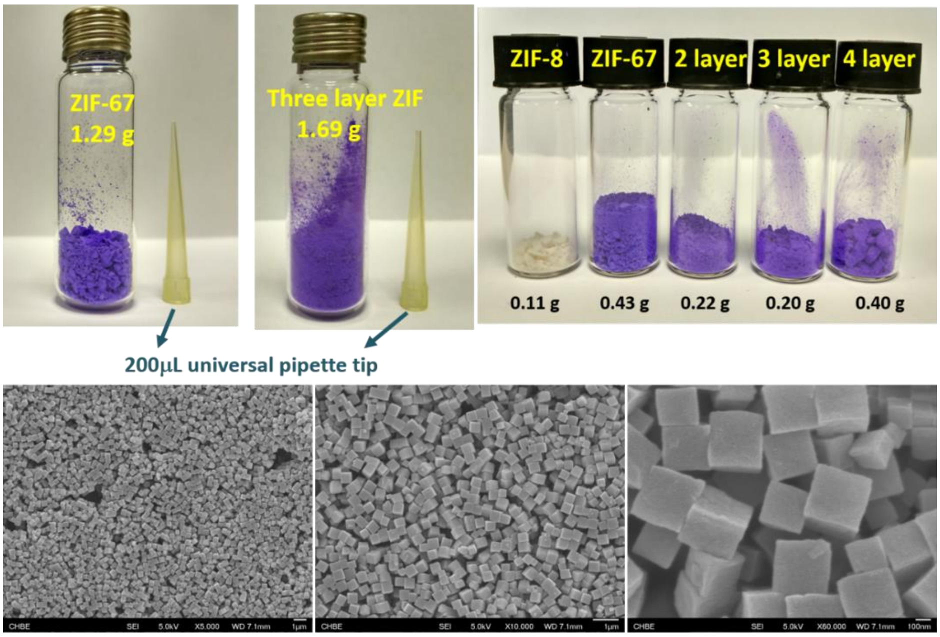

Supplementary Figure 14. Photographs of dry powders of ZIF-8, ZIF-67, and Matryoshka-type $(ZIFs@)_{n-1}ZIFs$ nanocubes prepared from "large-scale" synthesis via increasing the reaction scale by 100-fold; refer to Methods in the main text for more information.

Comments: The 2-layer ZIFs represents ZIF-67@ZIF-8, 3-layer ZIFs represents ZIF-67@ZIF-8@ZIF-67, and 4-layer ZIFs is denoted as ZIF-67@ZIF-8@ZIF-67@ZIF-8. The FESEM images (at different magnifications) at the bottom panel were obtained from the ZIF-67 sample from "large-scale" synthesis.

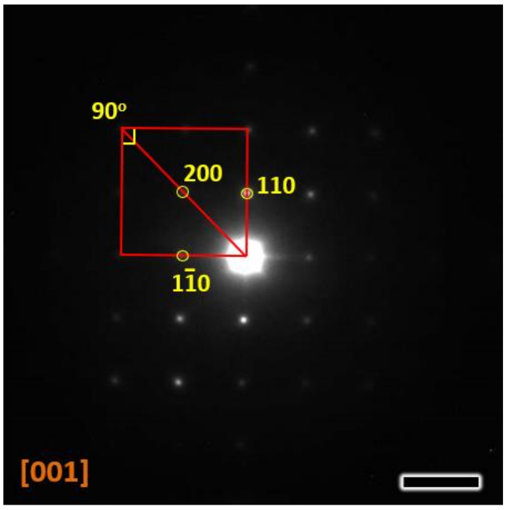

Supplementary Figure 15. A SAED pattern of cubic ZIF-67. Scale bar is 1 nm^{-1}.

Comments: The SAED pattern shows the [001] spot zone of a ZIF-67 nanocube, which confirms that the nanocube is faceted by the {100} family of crystal planes.

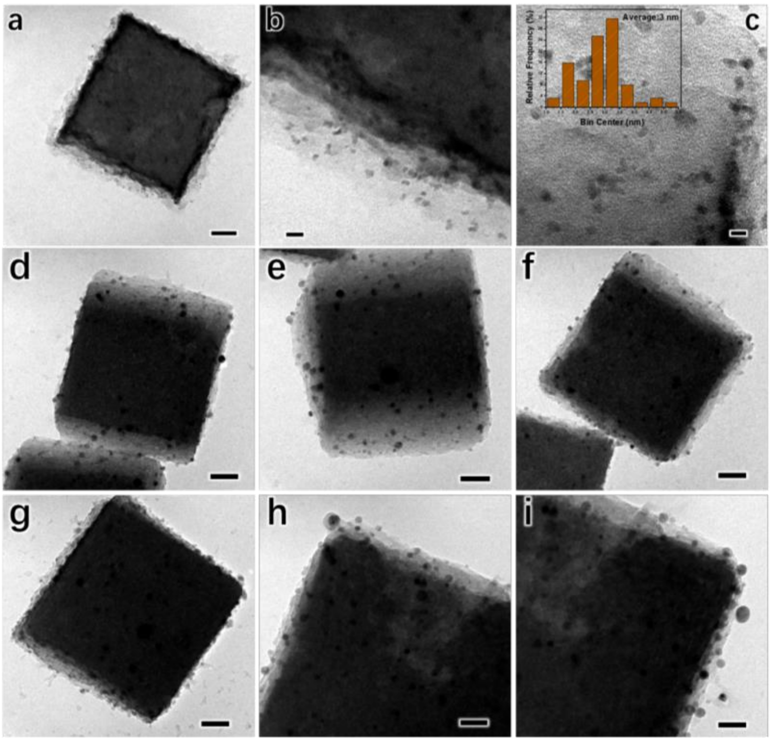

Supplementary Figure 16. (a-c) TEM images (at different magnifications) of ZIF-67/Pt. (d-i) TEM images (at different magnifications) of ZIF-67/Ag. The inset in (c) shows the size distribution histogram of Pt nanoparticles. Scale bars in (a-i) are 40 nm, 10 nm, 5 nm, 60 nm, 50 nm, 60 nm, 60 nm, 40 nm, and 30 nm, respectively.

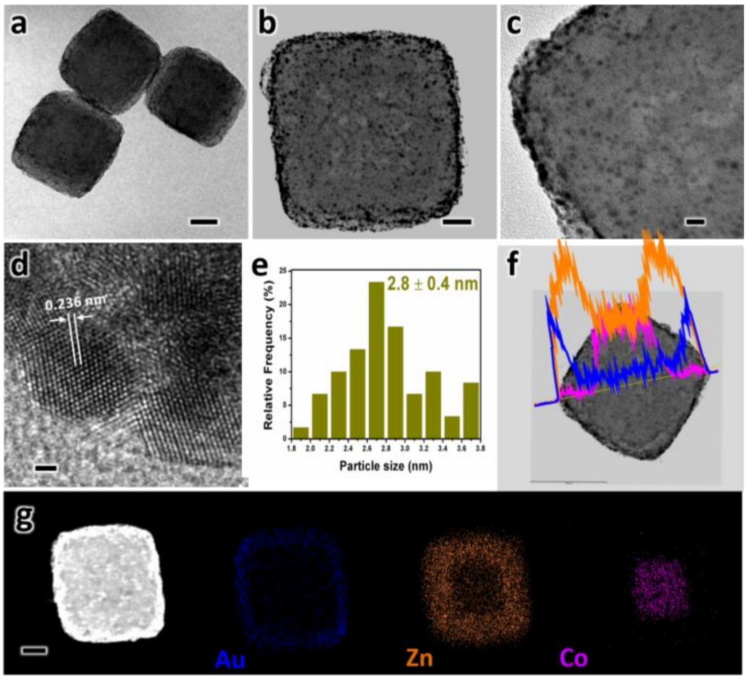

Supplementary Figure 17. Characterizations of ZIF-67@ZIF-8/Au. (a-d) TEM images, (e) the statistics of the particle size of the gold nanoparticles, (f) EDX elemental line scanning, and (g) EDX elemental maps. Scale bars in (a-d) are 60 nm, 30 nm, 10 nm, and 1 nm, respectively. Scale bar in (g) is 40 nm.

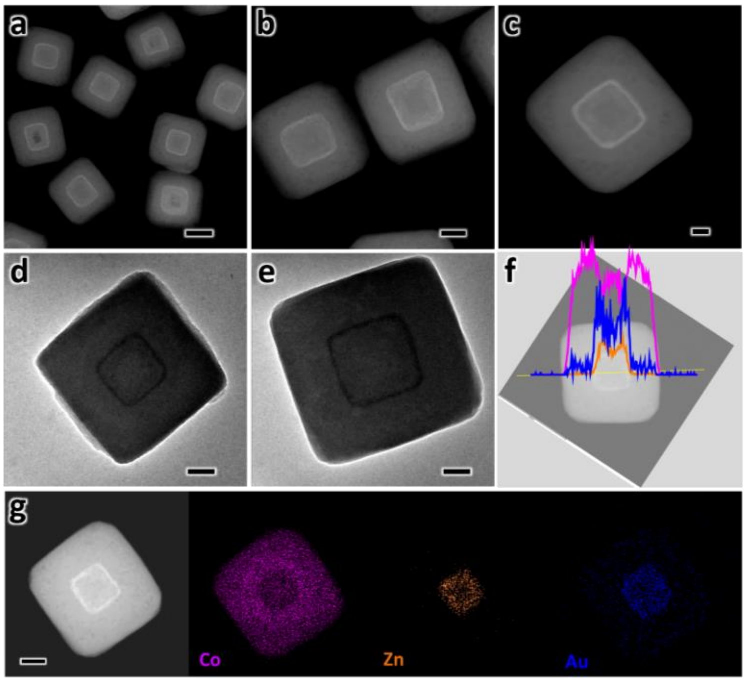

Supplementary Figure 18. Characterizations of ZIF-67@ZIF-8/Au@ZIF-67. The gold signal intensity in (f) was enlarged for 10 times in order to show more clearly the position of gold loading in Matryoshka-type $(ZIFs@)_{n-1}ZIFs$ nanocubes. Colour codes: pink = cobalt, blue = gold, and brown = zinc. Scale bars in (a-e) are 200 nm, 100 nm, 50 nm, 80 nm, and 60 nm, respectively. Scale bar in (g) is 100 nm.

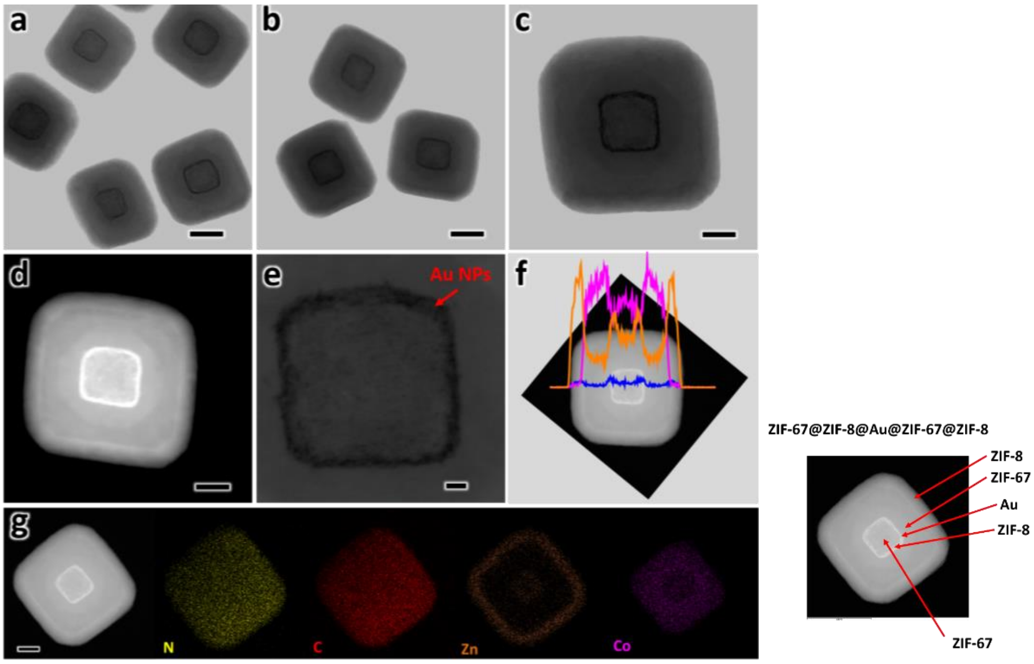

Supplementary Figure 19. Characterizations of ZIF-67@ZIF-8/Au@ZIF-67@ZIF-8. (a,b,c,e) TEM images, (d) STEM image, (f) EDX elemental line scanning, and (g) EDX elemental maps. Scale bars in (a-e) are 200 nm, 200 nm, 100 nm, 100 nm, and 20 nm, respectively. Scale bar in (g) is 100 nm.

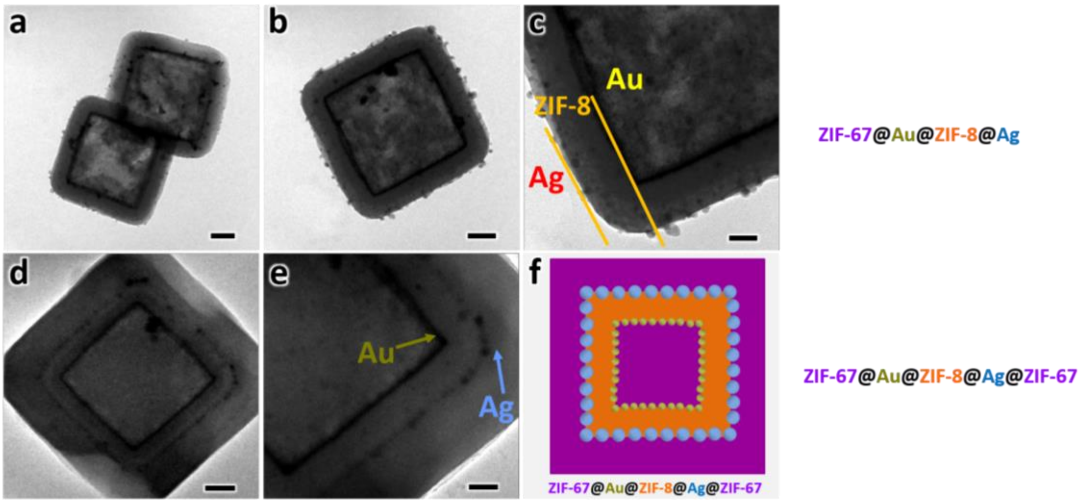

Supplementary Figure 20. TEM images (at different magnifications) and structural model of various ZIFs/metal nanocomposites: (a-c) ZIF-67/Au@ZIF-8@Ag, and (d-f) ZIF-67/Au@ZIF-8/Ag@ZIF-67. Scale bars in (a-e) are 100 nm, 80 nm, 40 nm, 100 nm, and 50 nm, respectively.

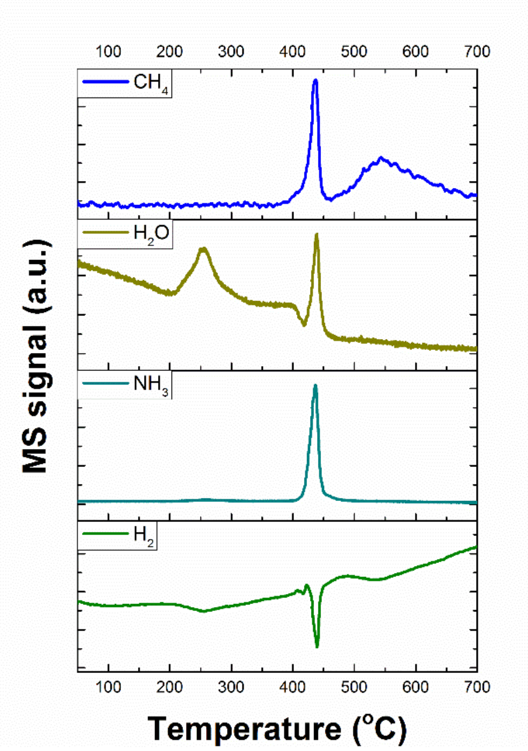

Supplementary Figure 21. TPR-MS profiles with temperature of ZIF-67 sample under a hydrogen stream. The evolution of ion with m/z of 44 (CO_2) was monitored but no signal was detected.

Comments: To monitor the thermal decomposition product of the ZIF-67/Pt under hydrogen gas, approximately 20 mg of the catalyst was heated in a quartz tube from 50°C to 700°C at a rate of 3 °C min^{-1} with an H_2 flow (60 mL min^{-1}). The gaseous products were detected by a mass spectrometer (MS). As shown, the detected gases generated in the hydrogenolysis process are CH_4, H_2O, and NH_3.

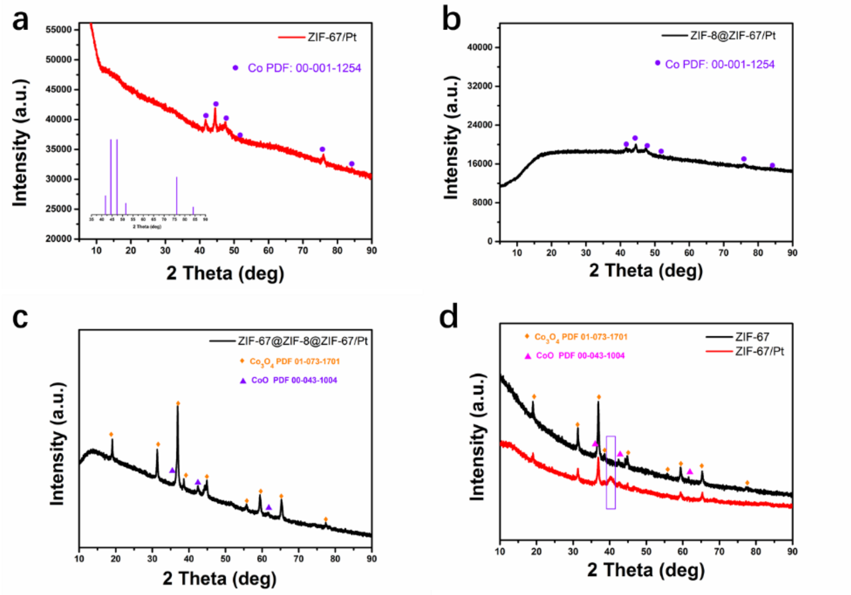

Supplementary Figure 22. (a) XRD pattern of the treated ZIF-67/Pt sample under hydrogen flowing at 180°C for 4 h, where metallic cobalt phase was found. Standard powder diffraction file (PDF) of metallic cobalt was provided in the figure (inset). (b) XRD pattern of the treated ZIF-8@ZIF-67/Pt sample under a hydrogen flow at 220°C for 4 h. (c) XRD pattern of the treated ZIF-67@ZIF-8@ZIF-67/Pt sample under hydrogen flowing at 300°C for 4 h. (d) XRD patterns of the ZIF-67 materials with Pt or without Pt after TGA tests (final temperature 800°C) under H_2/N_2 (4.2%/95.8%) flowing gas.

Comments: The result of this experiment indicates that ZIF-67 structure is easily decomposed if it was not separated from Pt, which is different from ZIF-67@ZIF-8/Pt configuration. The purple frame shown in the inset of the image (d) indicates the (111) diffraction peak of Pt (39.8 degrees). It was reported in the literature that even in the inert gas atmosphere, metallic cobalt was obtained.[1] Herein, the decomposed cobalt species would be reduced by the hydrogen. As shown, the metallic cobalt metal was further oxidized to cobalt oxide (such as CoO, Co_3O_4) during the sample treatment for XRD. Binary CoO and Co_3O_4 were found.

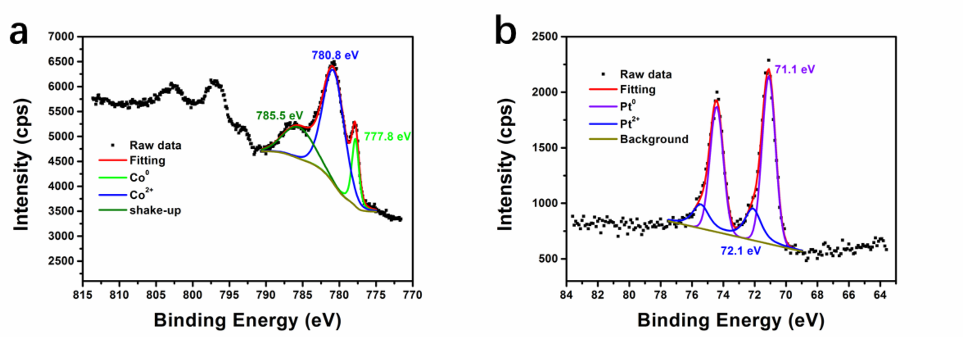

Supplementary Figure 23. XPS spectra in (a) Co 2*p* and (b) Pt 4*f* regions of the treated ZIF-67/Pt sample at 220°C for 4 h under flowing hydrogen.

Comments: The spectra in Co $2p_{3/2}$ region can be deconvoluted into three peaks corresponding to Co^0, Co^{2+}, and the shake-up satellite.[2] The presence of Co^{2+} is due to the oxidation of cobalt metal during sample preparation for XPS test. Moreover, as shown in Figure (b), most of the Pt species are in the metallic state.

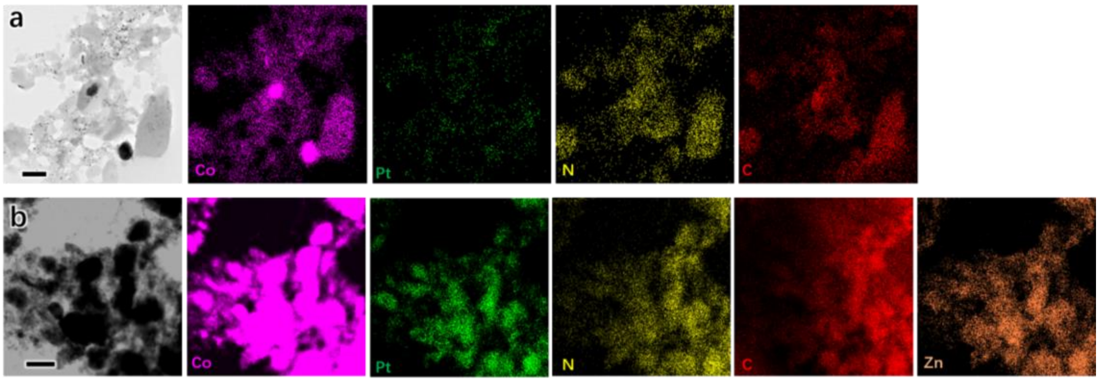

Supplementary Figure 24. EDX elemental maps of (a) ZIF-67/Pt and (b) ZIF-67@ZIF-8/Pt (ZIF-8 shell of 5 nm) after hydrogen flowing at 220ºC for 4 h. Scale bars in (a, b) are 100 nm and 300 nm, respectively.

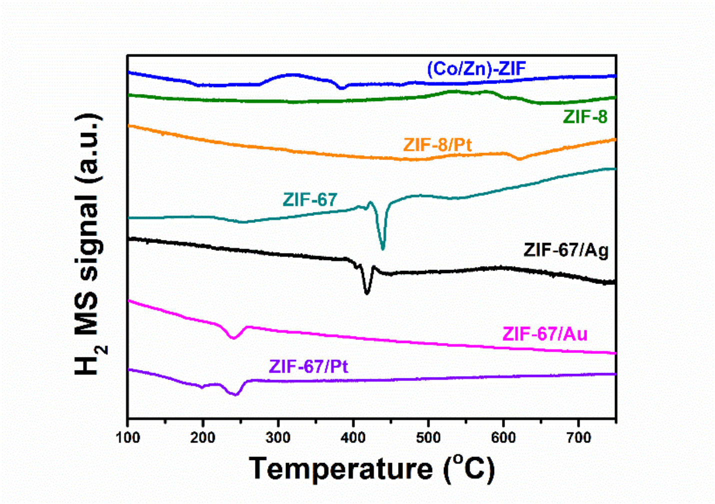

Supplementary Figure 25. TPR profiles of the different ZIFs and ZIFs/metal nanocomposite samples.

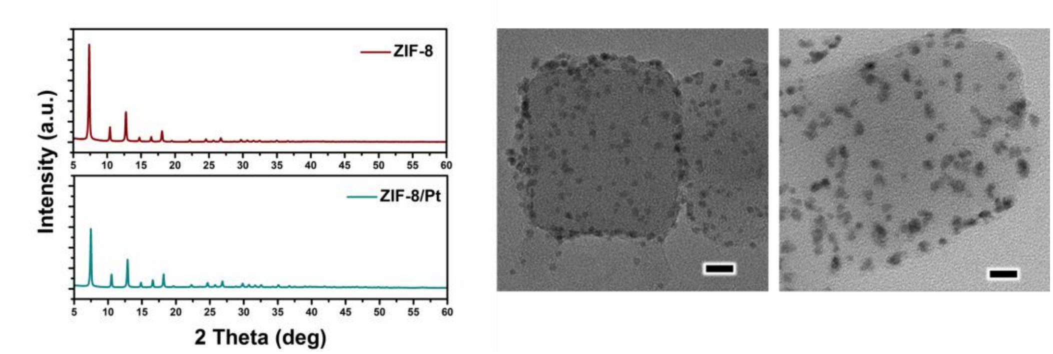

Supplementary Figure 26. XRD patterns and TEM images of the treated ZIF-8/Pt and pristine ZIF-8 samples under hydrogen flowing at 300ºC for 4 h. Scale bars in the two TEM images are 10 nm.

Comments: As shown, both ZIF-8 and ZIF-8/Pt are quite stable under hydrogen flowing at 300°C for 4 h. It means that both H_2 and H cannot reduce the Zn^{2+} ions in the ZIF-8 structure, which is quite different from Co^{2+} ions in ZIF-67 structure.

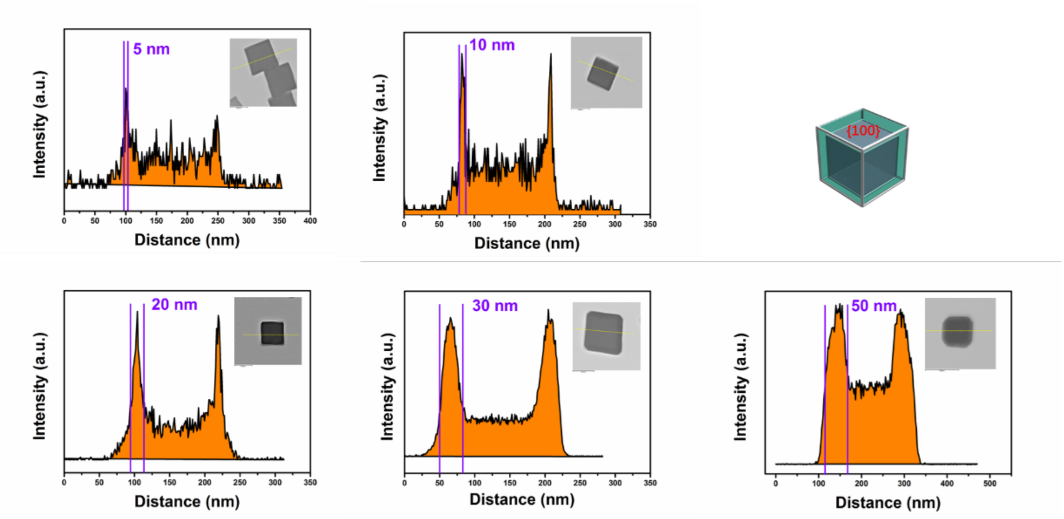

Supplementary Figure 27. EDX line scanning of ZIF-67@ZIF-8 with different shell thickness.

Comments: As shown, the thickness of ZIF-8 layer was adjusted as 5 nm, 10 nm, 20 nm, 30 nm, and 50 nm by tuning the synthesis parameters (*e.g.*, the amount of ZIF-67 core and the amount of zinc nitrate solution).

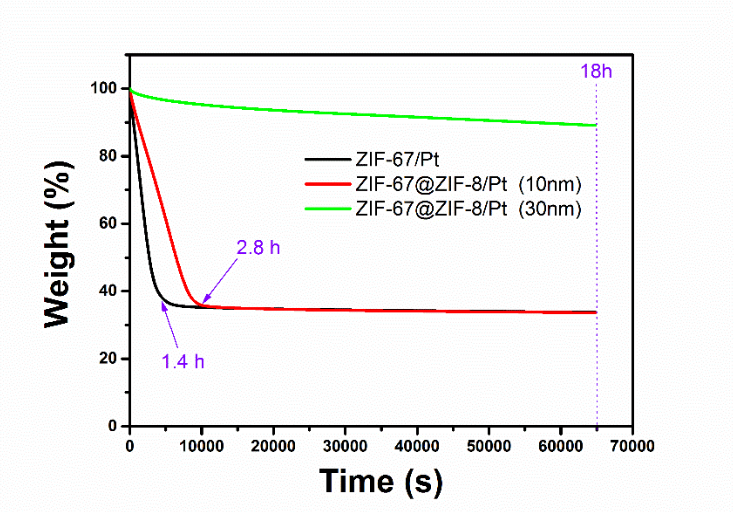

Supplementary Figure 28. Weight loss of the different samples at 260°C for 18 h under hydrogen flowing (4.6% H_2, 120 mL min^{-1}). The temperature was raised from room temperature to 260°C at a ramping rate of 3°C min^{-1} under the flowing of N_2 gas (120 mL min^{-1}). Sample amount 20 mg.

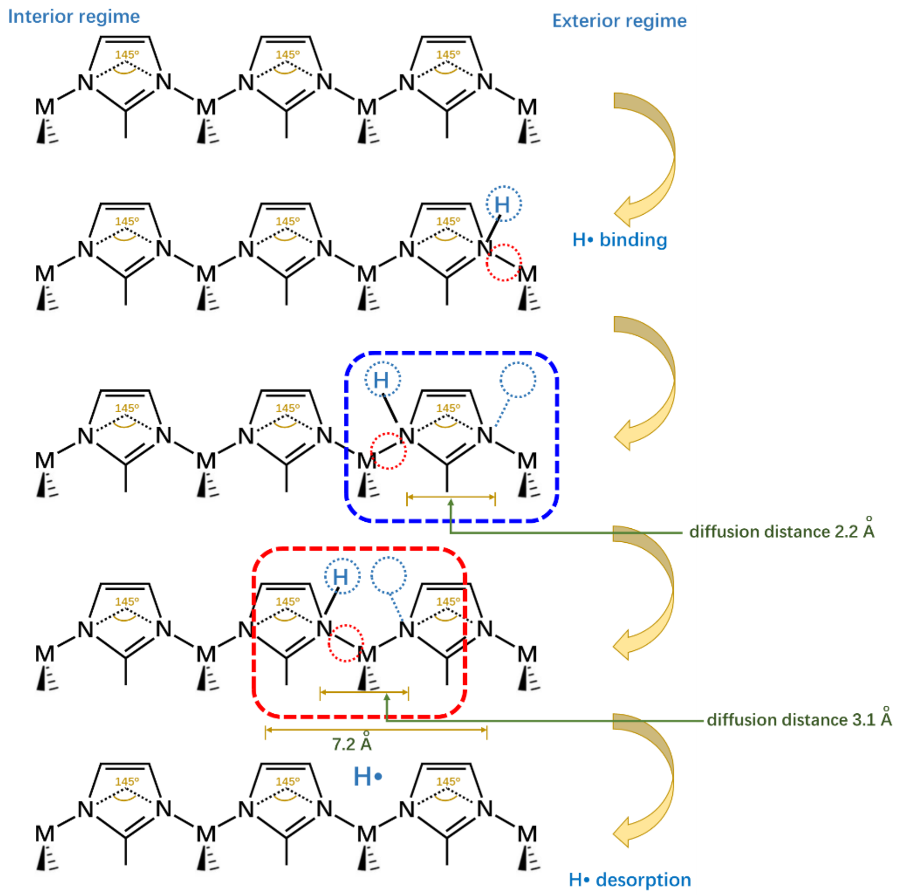

Supplementary Figure 29. A proposed mechanism for consecutive hydrogen atom migration on ZIFs, where the ZIFs structure provides transport tunnels for both electron and proton.

Comments: The above-proposed mechanism is an extension of a reported work on electron-proton mobility to the metal-organic frameworks. In that work,[3] the energy barrier (E_{act}) for combined electron-proton mobility on TiO_2 is between 0.6 and 0.7 eV, which is energetically more favourable than other possible diffusion mechanism (such as oxygen vacancies).[3] In Supplementary Figure 30, a migrating proton (an H atom can be viewed as a proton and e^- pair)[3] may prefer to bind with (negative) nitrogen of imidazolate linker, yielding a momentary N–H bond. Due to the adding proton, at the same time, the N–Zn bond is weakening or broken, and the electron (e^-) from the H atom is now associated with Zn^{2+} (i.e., the electron is now transferred to the conduction band of ZIF-8 which has a theoretical bandgap energy of E_g = 5.5 eV) to keep an overall charge balance. It is thought that this transition process would require a localized structural distortion, but this can be done easily as the structure of ZIFs is not rigid. Apparently, there are two main pathways for H atom transport: (i) ***intra-linker***: a proton moves within an imidazolate linker (Supplementary Fig. 29;[4] the diffusion distance is ca. 2.2 Å); and (ii) ***inter-linkers***: a proton transports between two imidazolate linkers (Supplementary Fig. 29; the distance of two adjacent imidazolate linkers is ca. 3.1 Å). We anticipate that future investigations by theoretical chemists will resolve the actual transport processes of H atoms.

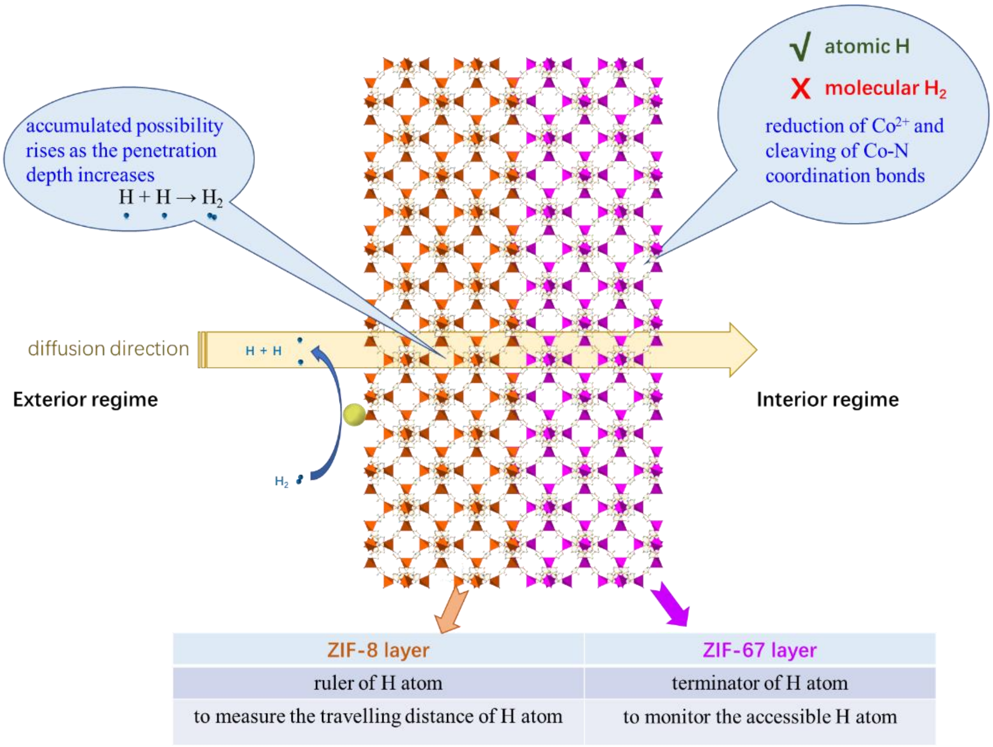

Supplementary Figure 30. A schematic illustration of hydrogen transportation through Matryoshka-type (ZIFs@)$_{n-1}$ZIFs nanocubes in which ZIF-67 serves as a sensitive detector for H atoms.

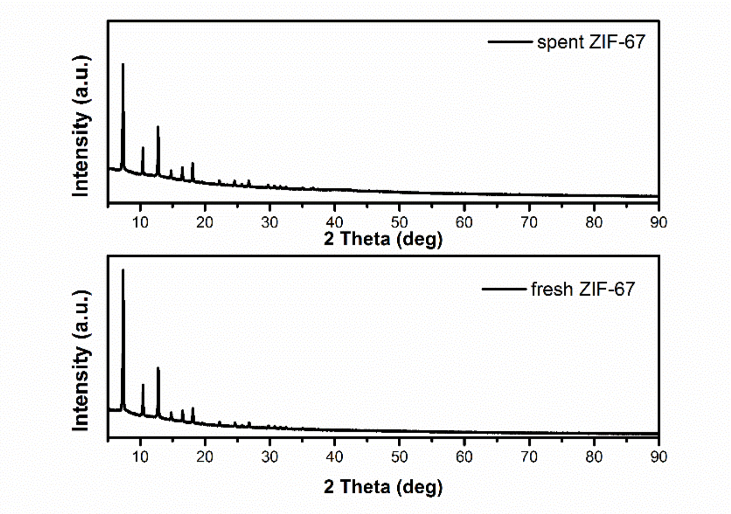

Supplementary Figure 31. Comparison of the XRD patterns of fresh and spent ZIF-67 after CO_2 hydrogenation reaction conducting at 240°C and 30 bar.

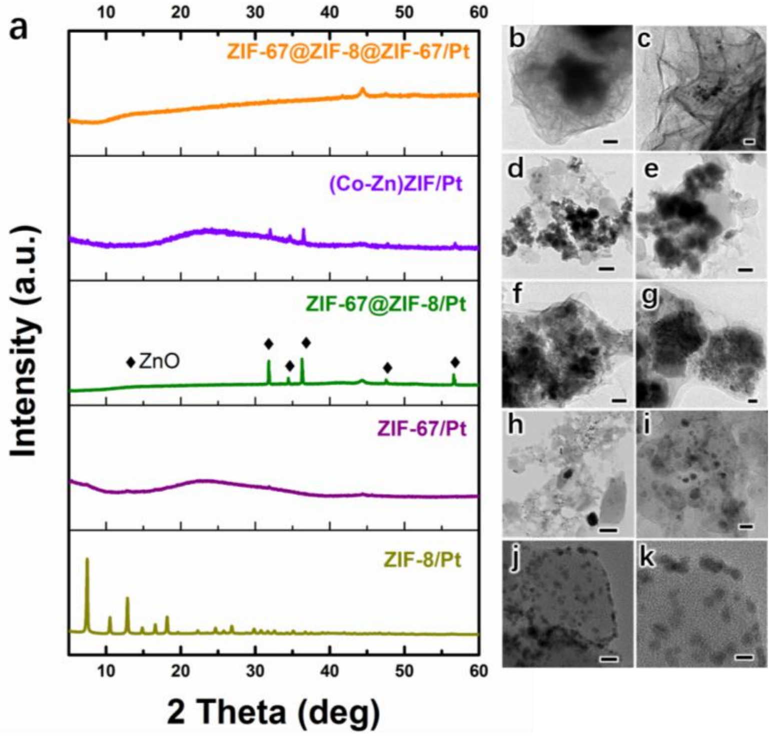

Supplementary Figure 32. XRD patterns and the corresponding TEM images of the spent catalysts after CO_2 hydrogenation reaction (220ºC and 30 bar). Scale bars in (b-k) are 40 nm, 10 nm, 100 nm, 50 nm, 20 nm, 10 nm, 100 nm, 10 nm, 10 nm, and 5 nm, respectively.

Comments: XRD patterns and TEM images of ZIF-8/Pt composite were analysed after the catalytic reaction conducting at 240ºC and 30 bar. In the ZIF-67@ZIF-8/Pt catalyst, the thickness of ZIF-8 layer was 20 nm.

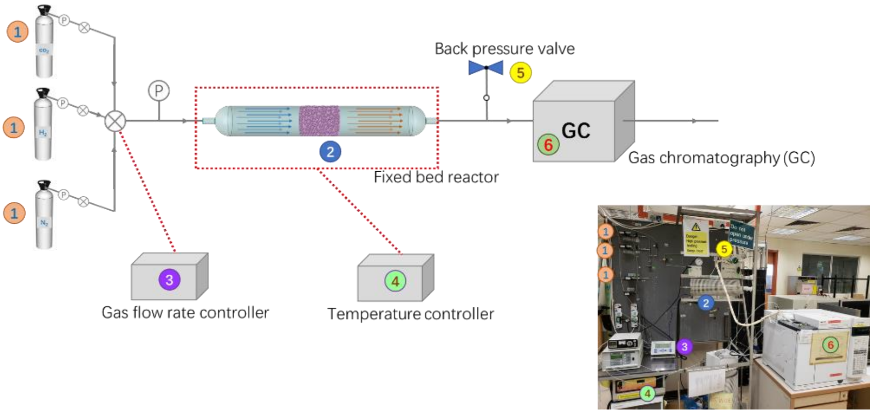

Supplementary Figure 33. Flowchart of the catalytic experimental set-up for testing ZIF-67 decomposition and the CO_2 hydrogenation performance. The right-bottom is a photograph of the experimental set-up for the reaction.

Supplementary Table 1. The surface atomic ratios of Zn/Co on the surface of some representative Matryoshka-type $(ZIFs@)_{n-1}ZIFs$ nanocubes (n = 4, 5, 6, and 7).

**Samples***	**Zn**	**Co**
ZIF-67@ZIF-8@ZIF-67@ZIF-8	100%	0%
ZIF-67@ZIF-8@ZIF-67@ZIF-8@ ZIF-67	0.62%	99.38%
ZIF-67@ZIF-8@ZIF-67@ZIF-8@ ZIF-67@ZIF-8	100%	0%
ZIF-67@ZIF-8@ZIF-67@ZIF-8@ ZIF-67@ZIF-8@ZIF-67	0.56%	99.44%

* Surface analysis data were measured by XPS technique. The overall configuration for A@B@C@D, etc., represents that "A" is located in the innermost part (core), "B" is the second layer, "C" is the third layer, and "D" is the fourth (the outmost) layer, etc.

Comments: As shown in Supplementary Table 1, no Co signal was found if the outmost layer of the composite is ZIF-8. Likewise, no Zn signal was observed if the outmost layer is ZIF-67. Therefore, the epitaxial growth of a new ZIFs layer is complete, indicating that the ZIFs in the shell could fully cover the cores.

Supplementary References

1. Chaikittisilp W, *et al.* Synthesis of Nanoporous Carbon–Cobalt-Oxide Hybrid Electrocatalysts by Thermal Conversion of Metal–Organic Frameworks. *Chem. - Eur. J.* **20**, 4217-4221 (2014).

2. Nabaho D, Niemantsverdriet JW, Claeys M, van Steen E. Hydrogen spillover in the Fischer–Tropsch synthesis: An analysis of platinum as a promoter for cobalt–alumina catalysts. *Catal. Today* **261**, 17-27 (2016).

3. Karim W, *et al.* Catalyst support effects on hydrogen spillover. *Nature* **541**, 68-71 (2017).

4. Bureekaew S, *et al.* One-dimensional imidazole aggregate in aluminium porous coordination polymers with high proton conductivity. *Nat. Mater.* **8**, 831-836 (2009).
